# Intermolecular carbene S–H insertion catalysed by engineered myoglobin-based catalysts†
†Electronic supplementary information (ESI) available: Experimental details, synthetic procedures, characterization data for reaction products and additional figures. See DOI: 10.1039/c5sc00080g



**DOI:** 10.1039/c5sc00080g

**Published:** 2015-02-09

**Authors:** Vikas Tyagi, Rachel B. Bonn, Rudi Fasan

**Affiliations:** a Department of Chemistry , University of Rochester , 120 Trustee Rd , Rochester , New York 14627 , USA . Email: fasan@chem.rochester.edu

## Abstract

The first example of a biocatalytic strategy for the synthesis of thioethers *via* an intermolecular carbene S–H insertion reaction is reported. Engineered variants of sperm whale myoglobin were found to efficiently catalyze this C–S bond forming transformation across a diverse set of aryl and alkyl mercaptan substrates and α-diazoester carbene donors, providing high conversions (60–99%) and high numbers of catalytic turnovers (1100–5400). Furthermore, the enantioselectivity of these biocatalysts could be tuned through mutation of amino acid residues within the distal pocket of the hemoprotein, leading to myoglobin variants capable of supporting asymmetric S–H insertions with up to 49% ee. Rearrangement experiments support a mechanism involving the formation of a sulfonium ylide generated upon attack of the thiol substrate to a heme-bound carbene intermediate.

## Abstract 2

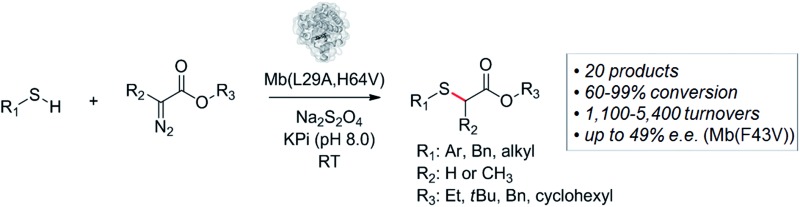
The first example of a biocatalytic strategy for the synthesis of thioethers *via* an intermolecular carbene S–H insertion reaction is reported.

## Introduction

The development of biocatalysts to execute synthetically valuable transformations not encountered in nature represents a key step toward the implementation of environmentally benign processes for organic synthesis.^1^ Catalytic methods for the selective formation of carbon–sulphur bonds are of particular interest as these bonds occur in a large number of natural and synthetic molecules with relevant biological activity, including an important fraction (∼20%) of marketed pharmaceuticals.^2^ A most direct approach for the construction of C–S bonds is through the insertion of carbenoid species, typically generated *via* the transition metal-catalyzed decomposition of diazo reagents, into the S–H bond of a mercaptan.^3^ In recent years, a number of transition metal catalysts, including copper,^4^ rhodium,^5^ iron^6^ and ruthenium^7^ complexes, have proven useful in the context of this reaction. Despite this progress, these systems are not devoid of limitations, which include moderate catalytic efficiencies and the requirement for slow addition of the diazo reagent to the reaction mixture in order to minimize undesirable side reactions. In addition, realizing the asymmetric insertion of carbenoids species into S–H bonds have proven particularly challenging, with low degrees of enantioselectivity (<25% ee) typically being observed^4b,5b,7b^ with a few notable exceptions.^4c,5d^ In contrast to the remarkable efforts made in the context of carbene S–H insertions using synthetic catalysts, there are no currently reports of natural or engineered enzymes capable of promoting this synthetically important transformation.

Our group and others have recently begun to explore the potential of cytochrome P450s and other heme-containing proteins as biocatalytic platforms for mediating ‘non-native’ carbene and nitrene transfer reactions.^1e,g,i,j,8^ In particular, we recently discovered that myoglobin and engineered variants thereof can promote the insertion of α-diazo-ester-derived carbenes into C=C double bonds (cyclopropanation) and into N–H bonds with high catalytic efficiency and in the case of the former, also with excellent levels of diastereo- and enantioselectivity.^1i,8b^ These results prompted us to investigate the reactivity and scope of these myoglobin-based catalysts toward carbene S–H insertion. Here, we report that engineered variants of sperm whale myoglobin can efficiently catalyze this C–S bond forming reaction across a broad range of aryl and aliphatic mercaptans as the substrates and across different α-diazo esters as the carbene donors. Furthermore, our studies show that the activity and enantioselectivity of these hemoprotein-based catalysts can be tuned *via* active site mutagenesis and provide first insights into the mechanism of this hemoprotein-mediated reaction.

## Results and discussion

In initial experiments, we investigated the activity of wild-type sperm whale myoglobin (Mb) toward catalyzing the insertion of ethyl α-diazoacetate (EDA, **2a**) into the S–H bond of thiophenol (**1**) in aqueous buffer (KPi, pH 8.0) and in the presence of sodium dithionite (Na_2_S_2_O_4_) as a reductant (Table 1). Promisingly, this reaction was found to lead to the desired S–H insertion product, ethyl α-(phenylthio)acetate (**3**), in 68% yield as determined based on GC analysis (entry 1, Table 1). In the course of optimization experiments, we established that nearly quantitative conversion of thiophenol to **3** (68 → 98%) along with higher catalytic turnovers (TON: 170 → 490), could be achieved using a two-fold excess of EDA over the thiol substrate at a catalyst loading of 0.2 mol% (entry 3, Table 1, Fig. S1†). Notably, comparable yields in this transformation have been obtained using transition metal complexes at 5- to 25-fold higher catalyst loadings (*i.e.*, 1–5 mol%).^4c,5b,d,7^ Furthermore, no formation of the dimerization byproducts, ethyl maleate and fumarate, was observed in these myoglobin-catalyzed reactions in spite of the presence of excess EDA and mixing of the reagents in a single addition (Fig. S1†). This result is in contrast with the slow addition protocols typically required to avoid this side reaction in the context of transition metal-catalyzed S–H insertion processes.^4c,5b,7^

**Table 1:** Catalytic activity of sperm whale myoglobin (Mb) for the carbene S–H insertion reaction with thiophenol and EDA^*a*^

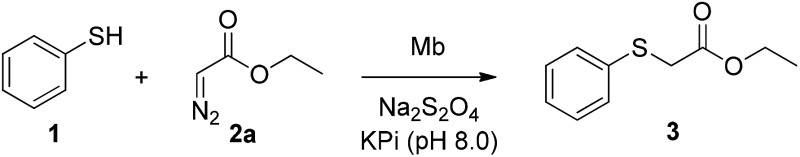						
Entry	[Mb] (mM)	[Na_2_S_2_O_4_] (mM)	[PhSH] (mM)	[EDA] (mM)	Conversion^*b*^	TON
1	0.02	10	10	5	68%	170
2	0.02	10	10	10	65%	325
3	0.02	10	10	20	98%	490
4	0.01	10	10	20	64%	640
5	0.005	10	10	20	49%	985
6	0.02	0.02	10	20	44%	220
7	0.02	—	10	20	26%	130

^*a*^Reaction conditions: 400 μL-scale reactions, 12 hours, room temperature, anaerobic conditions.

^*b*^Relative to the limiting reagent and as determined based on GC conversion using calibration curves with isolated **3**. Error is within 15%.

Time-course experiments showed that the Mb reaction with thiophenol and EDA was close to completion (94%) after 6 hours, with 68% of the S–H insertion product being formed within the first hour (Fig. S2†). These experiments also indicated that under these conditions the catalytic turnovers of the hemoprotein are limited by the concentration of thiophenol. By lowering the protein concentration, higher TON values were indeed observed (entries 4 and 5, Table 1), with Mb supporting a maximum of 985 turnovers at a catalyst loading of 0.05 mol%.

Investigation of the dependence of Mb activity on the reductant showed a decrease in TON as the sodium dithionite concentration was lowered, suggesting that ferrous Mb is the catalytically active form of the protein. Importantly, significant levels of catalytic activity were still maintained in the presence of stoichiometric amounts of sodium dithionite relative to the protein as compared to using an excess of reductant (220 *vs.* 492 TON; Table 1). Overall, these results are in line with our previous observations in the context of other Mb-catalyzed olefin cyclopropanation.^1*i*^ In contrast to the latter, however, appreciable Mb-dependent S–H insertion activity was observed also in the absence of reductant (130 TON; entry 7, Table 1). Even if not very efficiently for aromatic mercaptans, thiols have been shown to be able to reduce metmyoglobin to its ferrous form.^9^ Accordingly, the activity in the reductant-free reactions could be explained based on the formation of small amounts of the catalytically active ferrous Mb upon reaction with thiophenol. As observed for cyclopropanation and N–H insertion, molecular oxygen was found to supress Mb-dependent carbene S–H insertion reactivity, likely due to competition with the diazo reagent for binding to the heme center.

The observed S–H insertion activity supported by Mb is remarkable in light of the fact that thiols can readily coordinate the iron center in heme-containing proteins.^9a,b^ Efficient catalysis in these reactions is likely facilitated by the considerably lower affinity of these ligands for ferrous Mb as compared to metmyoglobin.^9*a*^ To examine possible substrate-dependent inhibitory effects, the reactions with Mb, thiophenol, and EDA were repeated using a thiophenol concentration of 40 and 80 mM. Interestingly, the Mb catalyst was found to remain active under these conditions, although a concentration-dependent reduction in catalytic efficiency also became apparent (62% and 42% relative activity, respectively, compared to 10 mM thiophenol), suggesting a certain degree of substrate inhibition at increasing thiol concentrations.

**Fig. 1:**
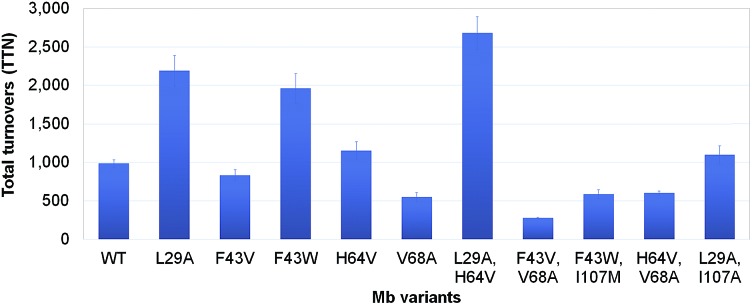
Total turnover numbers (TTN) supported by the different Mb variants for the conversion of thiophenol and EDA to **3**. Reaction conditions: 2.5 μM Mb variant, 10 mM PhSH, 20 mM EDA, 10 mM Na_2_S_2_O_4_ in KPi buffer (pH 8.0), 16 h.

**Fig. 2:**
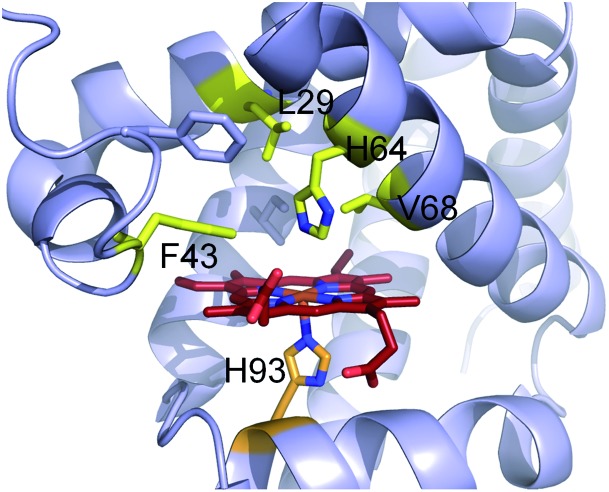
Active site of wild-type sperm whale myoglobin (pdb 1A6K). Residues targeted for mutagenesis are highlighted in yellow. The heme cofactor and proximal H93 ligand are colored in red and orange, respectively.

Encouraged by the results obtained with wild-type Mb, we turned our attention to identifying engineered Mb variants with enhanced reactivity toward S–H insertion. In previous studies, we found that mutations at the level of the distal pocket could improve the activity (and selectivity) of this hemoprotein toward carbene transfer reactions.^1i,8b^ Accordingly, a panel of Mb variants carrying one and two active site mutations were screened for their improved ability to convert thiophenol into **3** in the presence of EDA as determined based on total turnover numbers (TTN). Gratifyingly, a number of Mb variants were found to exhibit greatly increased (>2-fold) catalytic efficiency in this transformation (Fig. 1). In particular, the single mutant Mb(L29A) and double mutant Mb(L29A, H64V) emerged as the most promising catalysts, yielding 2190 and 2680 TTN, respectively, as compared to the 985 total turnovers supported by wild-type Mb. Interestingly, a similar activity-enhancing effect for the L29A mutation was noted also in the context of Mb-catalyzed carbene N–H insertion.^8*b*^ As discussed later, the beneficial effect of this substitution may be linked to its role in facilitating attack of the thiol (or amine) nucleophile to the heme-bound carbenoid intermediate. Noteworthy is also the additive effect of the H64V mutation, whose introduction leads to a comparable increase in TTN (∼20%) in both the wild-type Mb and the Mb(L29A) background. As judged based on analysis of the available crystal structure of the sperm whale myoglobin^10^ (Fig. 2), this mutation is likely to enhance the catalytic efficiency of Mb by increasing the accessibility of the heme pocket to the diazo ester and thiol reactants, as noted in the context of other non-native reactions supported by this protein.^1i,8b,d^ The initial rate for Mb(L29A, H64V)-catalyzed formation of the S–H insertion product **3** was determined to be 35 turnovers per minute, which is lower than that observed for related Mb variants toward N–H insertion (740 min^–1^)^8*b*^ and cyclopropanation (1000 min^–1^).^1*i*^

**Scheme 1:**
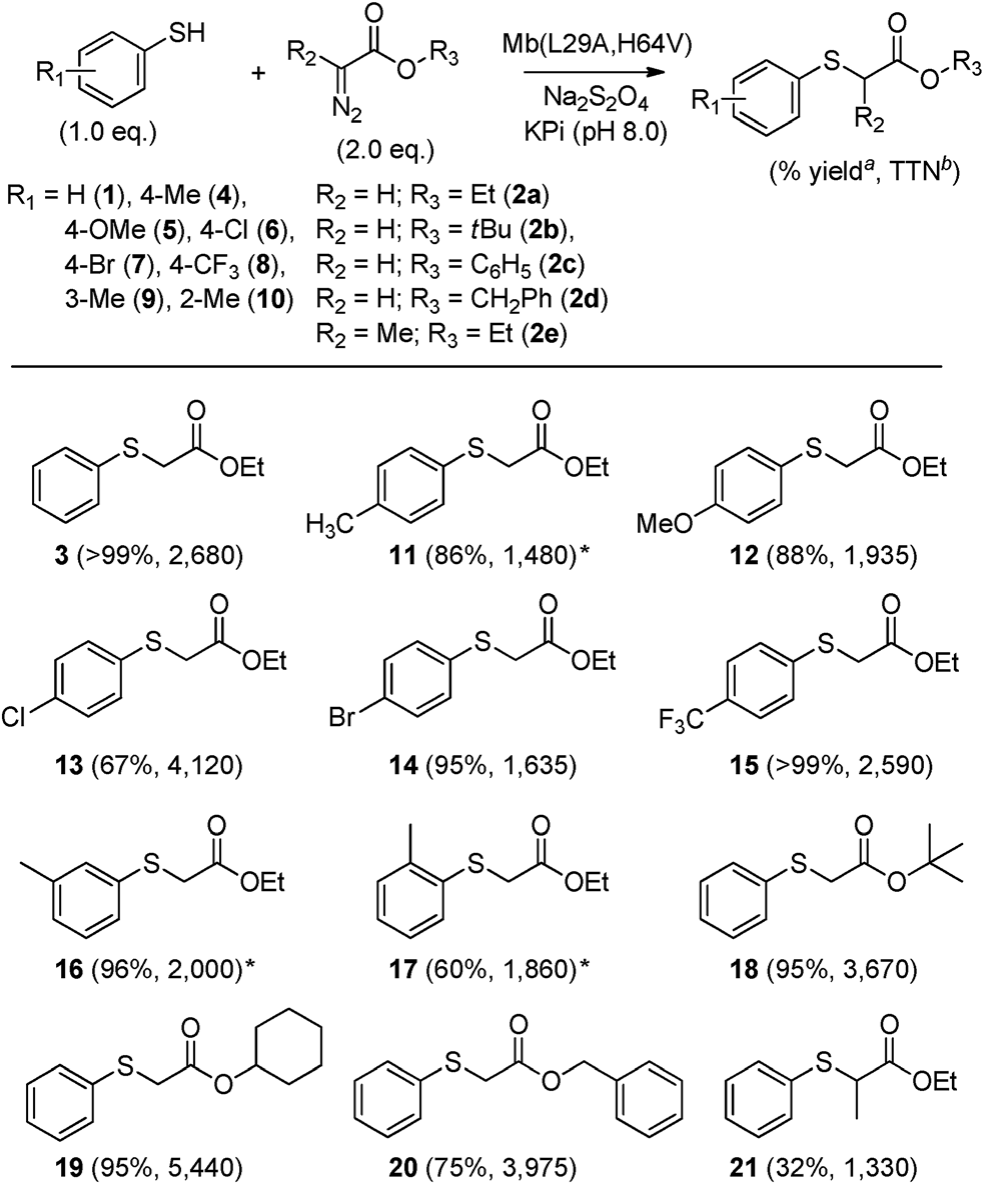
Yields and total turnover numbers (TTN) for Mb(L29A, H64V)-catalyzed carbene S–H insertion with various aryl mercaptans and α-diazo esters. Reaction conditions: 10 mM thiol, 20 mM EDA, 10 mM Na_2_S_2_O_4_ with (a) 20 μM (0.2 mol%) and (b) 2.5 μM (0.025 mol%) hemoprotein, 16 hours. * Buffer added with 20% (v/v) methanol.

Having identified Mb(L29A, H64V) as the most promising Mb-based catalyst for S–H insertion, further experiments were carried out to explore its scope across different aryl mercaptans (Scheme 1). To this end, variously substituted thiophenols (**4–10**) were subjected to Mb(L29A, H64V) catalysis (0.02 mol%) in the presence of EDA (**2a**). Notably, high to quantitative conversion (67–99%) to the desired S–H insertion products (**11–15**) were obtained starting from the *para*-substituted thiophenol derivatives **4–8**, showing that electron-donating and electron-withdrawing substituents at this position are equally well tolerated by the protein catalyst. Similar results were obtained with the *meta*- and *ortho*-substituted thiophenols **9** and **10**, respectively, although a certain influence of the *ortho* substitution on the efficiency of the reaction was also evident (60% *vs.* 86–96% conversion for **17** *vs.* **11** and **16**).

To further investigate the reactivity scope of Mb(L29A, H64V), the reactions with thiophenol were then performed in the presence of different types of α-diazo ester reagents, namely *tert*-butyl (**2b**), cyclohexyl (**2c**), and benzyl α-diazoacetate (**2d**). Importantly, formation of the respective S–H insertion products **18**, **19**, and **20** in 75–95% yields demonstrated the high degree of tolerance of the Mb-derived catalyst toward substitutions at the level of the ester group of the diazo reagent. Albeit in more moderate yield (32%), successful synthesis of **21** indicated that Mb(L29A, H64V) can also accept the α-substituted ethyl α-diazopropanate (**2e**) as a carbene donor. Altogether, the experiments outlined in Scheme 1 demonstrated the broad scope of the Mb(L29A, H64V) catalyst across different aryl thiol substrates and diazo reagents. Furthermore, repeating these reactions under low catalyst loading conditions (0.025 mol%) showed that Mb(L29A, H64V) support TTN values in excess of 1300 in each case, yielding over 4100 and 5400 TTN in the reactions with *p*-chloro-thiophenol and EDA (product **13**) and with thiophenol and cyclohexyl α-diazoacetate (product **19**), respectively. These catalytic efficiencies are one to two orders of magnitude higher than those reported for similar S–H insertion reactions with transition metal catalysts.^4c,5b,d,7^

In order to assess the scalability of these Mb-catalyzed transformations, the synthesis of ethyl α-(phenylthio)acetate (**3**) from **1** and **2a** was carried out at a larger scale (∼11 mg) thiophenol, 0.2 mol% Mb(L29A, H64V). Successful isolation of 13.2 mg of **3** from this reaction in 67% isolated yield thus demonstrated the potential utility of these Mb-mediated reactions for synthetic purposes.

To determine whether the scope of Mb(L29A, H64V)-mediated S–H insertion could be extended to non-aromatic thiols, tests with benzylic and alkyl mercaptans as the substrates were carried out. In the presence of EDA, Mb(L29A, H64V) was found to readily functionalize benzyl mercaptan (**22**), substituted benzyl mercaptan derivatives (**23–25**), and alkyl mercaptans such as cyclohexanethiol (**26**) and octane-1-thiol (**27**), providing conversions in the range of 30–50% and supporting between 930 and 2550 total turnover numbers (entries 1–6, Table 2). Given the clear advantage of using α-diazo esters with large alkyl groups (i.e., **2e** or **2d**) toward improving the TTN in the insertion reactions with thiophenol (Scheme 1), the Mb(L29A, H64V)-catalyzed transformations of benzyl (**22**) and cyclohexanethiol (**26**) were carried out also in the presence of **2d**. Gratifyingly, higher yields (83–99%) and total turnovers (TON: 3–4600) were obtained in both cases (Table 2), further evidencing the good match between the Mb catalyst and these carbene donors.

**Table 2:** Substrate scope and catalytic activity of Mb(L29A, H64V) toward carbene S–H insertion in the presence of different alkyl mercaptans and α-diazo esters

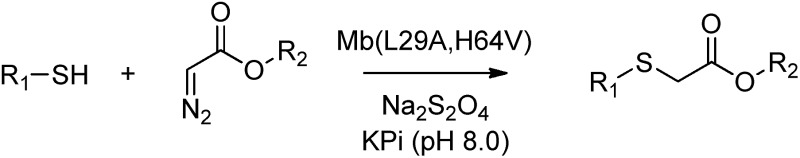					
Entry	R_1_	R_2_	Product	Conv.^*a*^	TTN^*b*^
1	Bn (**22**)	Et	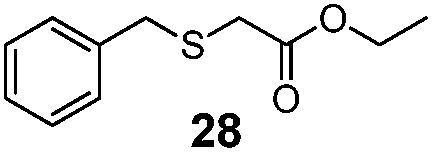	36%	2550
2	(4-Me)PhCH_2_ (**23**)	Et	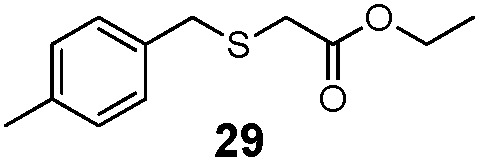	51%	2060
3	(4-OMe)PhCH_2_ (**24**)	Et	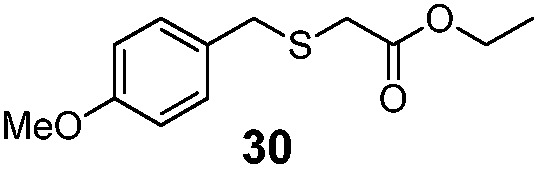	49%	2550
4	(4-Cl)PhCH_2_ (**25**)	Et	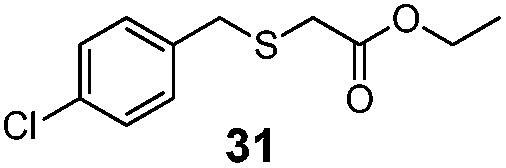	30%	930
5	C_6_H_11_ (**26**)	Et	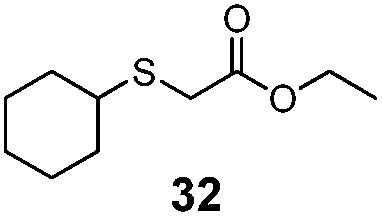	30%	1100
6	*n*-Octyl (**27**)	Et	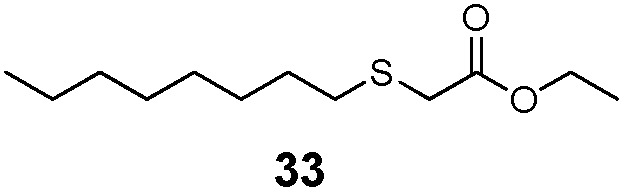	51%	1730
7	Bn (**22**)	Bn	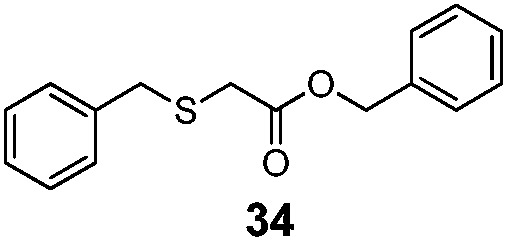	83%	3050
8	C_6_H_11_ (**26**)	Bn	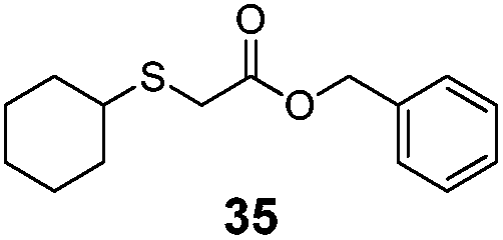	>99%	4620

^*a*^Reaction conditions: 10 mM thiol, 20 mM diazo ester, 20 μM Mb(L29A, H64V) (0.2 mol%), 10 mM Na_2_S_2_O_4_ in oxygen-free phosphate buffer (pH 8.0), 16 hours.

^*b*^Reaction conditions: same as (a) but using 0.025 mol% protein (2.5 μM).

The development of catalytic systems for asymmetric carbene S–H insertions has proven remarkably difficult, with only low levels of enantioselectivity being observed in most cases (8–23% ee).^4b,5b,7b^ In this area, significantly better results (70–85% ee) have been more recently achieved by Zhou and coworkers using chiral spiro bis(oxazoline)-copper complexes, although these protocols involve high catalyst loadings (5 mol%) along with additives (6 mol% NaB(Ar^F^)_4_).^4*c*^ Interestingly, the reaction of ethyl α-diazo-propanoate with thiophenol in the presence of wild-type Mb or Mb(L29A, H64V) showed that neither of these proteins was capable of providing chiral induction in the S–H insertion reaction (entries 1 and 2, Table 3). Screening of the panel of Mb active-site variants revealed however that both Mb(F43V) and Mb(F43V, V68A) showed appreciable enantioselectivity in this transformation (21–22% ee, entries 3 and 5 in Table 3; Fig. S3†). Since Mb(V68A) exhibited only 6% ee, the beneficial effect in terms of enantioselectivity can be mainly attributed to the substitution at the level of Phe43, which is located in close proximity to the heme cofactor (Fig. 2).

**Table 3:** Enantioselectivity of myoglobin (Mb) and variants thereof for the carbene S–H insertion reaction in the presence of ethyl α-diazopropanoate (**2e**)^*a*^

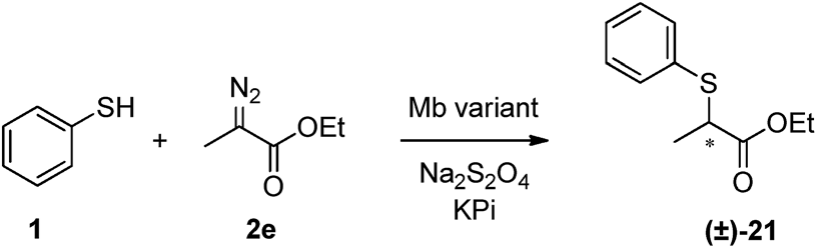						
Entry	Catalyst	[PhSH] (mM)	[EDA] (mM)	pH/temp.	ee^*b*^	TON
1	Mb	10	20	8.0/r.t.	0	105
2	Mb(L29A, H64V)	10	20	8.0/r.t.	0	160
3	Mb(F43V)	10	20	8.0/r.t.	22%	160
4	Mb(V68A)	10	20	8.0/r.t.	6%	95
5	Mb(F43V, V68A)	10	20	8.0/r.t.	21%	145
6	Mb(F43V)	10	10	8.0/r.t.	29%	80
7	Mb(F43V)	10	10	7.0/r.t.	32%	60
8	Mb(F43V)	10	10	7.0/4 °C	49%	75

^*a*^Reaction conditions: 400 μL-scale reactions, 20 μM protein, 10 mM Na_2_S_2_O_4_ 12 hours, room temperature, anaerobic conditions.

^*b*^As determined based on chiral gas chromatography using racemic standards for calibration.

Further improvements in the enantioselectivity of the Mb(F43V)-catalyzed insertion reaction could be then achieved through optimization of the thiol : diazo ester ratio and other reaction parameters such as pH and temperature (entries 6–8, Table 3). Under optimal conditions (**1** : **2e** in 1 : 1 ratio, pH 7.0, 4 °C), the S–H insertion product **21** was obtained with an enantiomeric excess of 49% (Fig. S3†), which corresponds to the highest enantioselectivity ever reported with a single-catalyst system and in the absence of exogenous additives.^4c,5d^ Taken together, these results support the amenability of the Mb scaffold to promote asymmetric carbene S–H insertions as well as the possibility to tune this property *via* active site engineering.

**Scheme 2:**
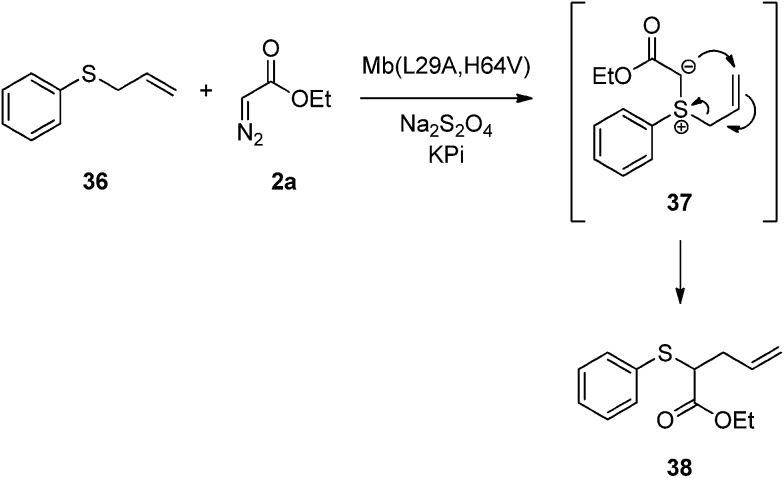
Mb(L29A, H64V)-catalyzed rearrangement of allyl(phenyl)sulfane **36** in the presence of ethyl α-diazoacetate (**2a**).

Depending on the nature of the transition metal catalyst, the insertion of carbenoids into Y–H bonds (where Y = O, N or S) has been proposed to involve either a concerted^11^ or stepwise mechanism,^6a,12^ the latter proceeding through a oxonium/ammonium/sulfonium ylide formed upon attack of the Y–H nucleophile to a metal carbenoid intermediate. To shed light into the mechanism of Mb-catalyzed carbene S–H insertion, trapping of the putative sulphur ylide intermediate in the reaction with Mb(L29A, H64V), thiophenol, and EDA, was first attempted *via* addition of diethyl azadicarboxylate (DEAD), as this approach have proven useful to reveal the occurrence of a stepwise mechanism in other systems.^6a,13^ However, only the conjugate addition product generated *via* direct attack of thiophenol to DEAD was observed. As an alternative approach, allyl(phenyl)sulfane **36** was made react with Mb(L29A, H64V) in the presence of EDA (Scheme 2). Interestingly, the only species generated in this reaction was the rearrangement product **38** (TON: 390), whose formation is consistent with the [2,3]-sigmatropic rearrangement of a sulfonium ylide intermediate^6a,14^ (**37**, Scheme 2). While this C–C forming transformation has synthetic value on its own right,^15^ its most relevant implication in the context of this work is that of supporting the occurrence of a step-wise mechanism for Mb-catalyzed carbene S–H insertion.

**Scheme 3:**
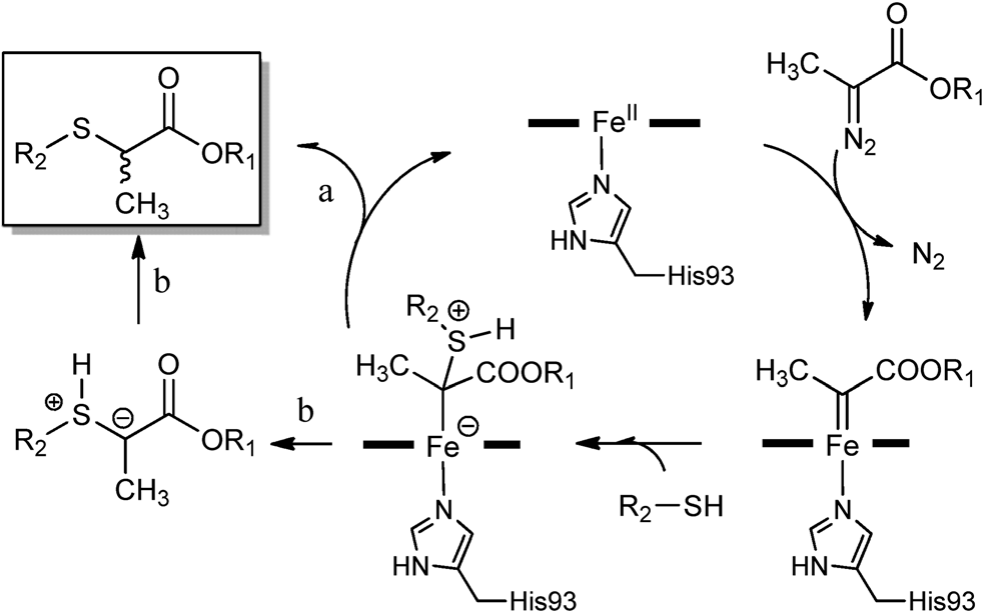
Proposed mechanism for myoglobin-catalyzed carbene S–H insertion.^17^

Based on these results and our previous studies,^1*i*^ we propose a mechanism which involves the initial formation of a heme-bound carbene intermediate upon reaction of ferrous myoglobin with the α-diazo ester, followed by nucleophilic attack of the thiol to give a sulphur ylide (Scheme 3). The S–H insertion product would then ensue *via* a proton transfer to the latter intermediate either prior to (path ‘a’) or after dissociation from the heme (path ‘b’). Indirect evidence that the ylide may undergo protonation at the carbon center while remaining (at least in part) bound to the heme derives from the observed influence of active site substitutions on the enantioselectivity of the reaction in the presence of the α-substituted diazo ester **2e** (Table 3). Indeed, complete dissociation of the ylide from the metal center would produce a racemic product. The latter process has been identified as a major factor contributing to the low enantioselectivity exhibited by transition metal catalysts in S–H insertion reactions.^4d,16^ Based on these considerations, it is possible that, in addition to affecting the selectivity of the proton transfer step within the distal pocket, the F43V substitution may potentially improve the enantioselectivity of the reaction also by disfavouring dissociation of the sulphur ylide from the heme center. An alternative, albeit not exclusive scenario would entail that the selectivity-determining step is associated with the addition of the thiol nucleophile to the heme–carbene complex. In this case, the role of the protonation step in influencing the enantioselectivity of the reaction would be dependent upon the relative configurational stability of the heme bound ylide.

While further studies are required to elucidate these steps in more details, the mechanistic model of Scheme 3 can already provide a plausible explanation for the effect of the L29A mutation toward enhancing the catalytic activity of the hemoprotein (Fig. 1). Given its location in the active site (Fig. 2), this substitution is expected to expand the distal cavity above the heme, thereby better accommodating the aryl/alkyl group of the thiol substrate after attack to the electrophilic carbene moiety group (R_2_ group, Scheme 3). Consistent with this hypothesis, the catalytic activity of wild-type Mb on the relatively bulky cyclohexanethiol (**26**) was found to be only ∼10% that of the L29A-containing variants. In that regard, it is also instructive to note how the L29A mutation was beneficial for Mb-catalyzed N–H insertion,^8*b*^ in which a mechanism similar to that of Scheme 3 could be operative,^18^ but not for Mb-catalyzed cyclopropanation, for which experimental data support a concerted carbene transfer step and a different mode of approach of the olefin to the heme-bound carbenoid.^1*i*^ Finally, it is worth mentioning that, unlike in the case of the cyclopropanation reactions,^1*i*^ a Hammett analysis of the relative rates of S–H insertion for different *para*-substituted thiophenol derivatives (*i.e.*, **11–13**, **15**) *versus* thiophenol did not yield a linear correlation for the corresponding plot of log(*k*_X_/*k*_H_) against *σ* (or *σ* ^+^) (Fig. S4†). While multiple factors could contribute to this phenomenon, these results remain consistent with the mechanistic scenario outlined above. Indeed, according to it, the aryl ring of these substrates is expected to come in close proximity to residues within the distal pocket of the protein. As such, both steric and electronic effects associated with the nature of the *para* substituent are expected to influence the rate of these reactions, thereby possibly contributing to the nonlinearity of the Hammett plot.

## Conclusions

Myoglobin has represented an attractive scaffold for biocatalyst development.^19^ This work demonstrates that engineered Mb variants constitute efficient systems for promoting carbene S–H insertion reactions, providing the first example of a biocatalyst capable of supporting this valuable transformation. These Mb-based catalysts were found to offer high catalytic activity (1100–5400 TON) across a wide range of aryl and alkyl mercaptan substrates as well as across different α-diazo esters as carbene precursors. Their potential utility for synthetic applications is further supported by proof-of-principle demonstrations of their ability to catalyze asymmetric S–H insertions and of the scalability of these reactions. Initial insights into the mechanism of this reaction were gleaned through the present studies, which can provide a basis for further optimization of the activity and selectivity of these biocatalysts. Finally, the ability of these hemoproteins to catalyze the [2,3]-sigmatropic rearrangement of allyl sulphides (**36** → **38**) is another notable finding of this work and the scope of this transformation is currently under investigation in our laboratory.

## References

[cit1] Collot J., Gradinaru J., Humbert N., Skander M., Zocchi A., Ward T. R. (2003). J. Am. Chem. Soc..

[cit2] (a) List of 2012 top 200 marketed drugs by sales: http://www.pharmacytimes.com/publications/issue/2013/July2013/Top-200-Drugs-of-2012.

[cit3] (a) Doyle M. P. and Ye T., Modern catalytic methods for organic synthesis with diazo compounds, Wiley, New York, 1998.

[cit4] Yates P. (1952). J. Am. Chem. Soc..

[cit5] Paulissen R., Hayez E., Hubert A. J., Teyssie P. (1974). Tetrahedron Lett..

[cit6] Aviv I., Gross Z. (2008). Chem.–Eur. J..

[cit7] Galardon E., LeMaux P., Simonneaux G. (1997). J. Chem. Soc., Perkin Trans. 1.

[cit8] Wang Z. J., Peck N. E., Renata H., Arnold F. H. (2014). Chem. Sci..

[cit9] Shimizu T., Nozawa T., Hatano M. (1976). Biochim. Biophys. Acta.

[cit10] Vojtechovsky J., Chu K., Berendzen J., Sweet R. M., Schlichting I. (1999). Biophys. J..

[cit11] Davis F. A., Yang B., Deng J. (2003). J. Org. Chem..

[cit12] Lu C. D., Liu H., Chen Z. Y., Hu W. H., Mi A. Q. (2005). Org. Lett..

[cit13] Huang H., Wang Y., Chen Z., Hu W. (2005). Adv. Synth. Catal..

[cit14] Kido F., Abiko T., Kato M. (1992). J. Chem. Soc., Perkin Trans. 1.

[cit15] Li A. H., Dai L. X., Aggarwal V. K. (1997). Chem. Rev..

[cit16] Consistent with this scenario, the use of a chiral Brønsted acid to intercept the free ylide has proven effective in greatly enhancing the enantioselectivity of carbene S–H insertions.5*d*

[cit17] The present scheme features the thiol nucleophile in its protonated form, which is predominantly populated by the alkyl and benzylic mercaptan substrates (p*K* _a_ > 8.5–9) under the applied conditions (pH = 8.0). Given that the aryl mercaptan substrates are largely deprotonated at this pH (p*K* _a_ < 6.5), the participation of an analogous mechanism in which a thiolate (R_2_–S^–^) acts as the nucleophile in place of the protonated thiol (R_2_–SH) can be also envisioned

[cit18] Mechanistic similarities between N–H insertion and S–H insertion reactions have been found in the context of other catalytic systems.6*a*

[cit19] Ozaki S., Matsui T., Watanabe Y. (1997). J. Am. Chem. Soc..

