# Supplementary material for: Intermolecular carbene S–H insertion catalysed by engineered myoglobin-based catalysts
Source: Chem Sci. 2015 Feb 9;6(4):2488–94. doi: 10.1039/c5sc00080g (PMC4474517; doi:10.1039/c5sc00080g)
Supplement: Supplementary file 1 [file SC-006-C5SC00080G-s001.pdf]

Electronic Supplementary Material (ESI) for Chemical Science.
This journal is © The Royal Society of Chemistry 2015

Supporting Information for

Intermolecular carbene S-H insertion catalysed by engineered myoglobin-based catalysts

VikasTyagi, Rachel B. Bonn, and Rudi Fasan*

Department of Chemistry, University of Rochester, 14627 Rochester, New York, USA

Correspondence should be addressed to R.F. (fasan@chem.rochester.edu)

Table of contents:

Figure S1-S4	Page S2-S6
Experimental Procedures	Pages S7-S10
Synthetic Procedures	PageS11-S20
References	Page S21
NMR Spectra	Page.S21-S41

Figure S1: Representative GC chromatogram corresponding to the reaction of thiophenol and EDA in the presence of wild-type Mb as the catalyst. The peaks corresponding to the S−H insertion product, α-(phenylthio)acetate (**3**), and the internal standard are labelled. Thiophenol elutes at 2.42 min and is completely consumed in the reaction. Trace amounts of diphenyldisulfide (labeled with *) are observed in the reaction mixture. Reaction conditions: 20 μM Mb (0.2 mol%), 10mM thiophenol, 20 mM EDA, 10 mM dithionite in oxygen-free phosphate buffer (pH 8.0).

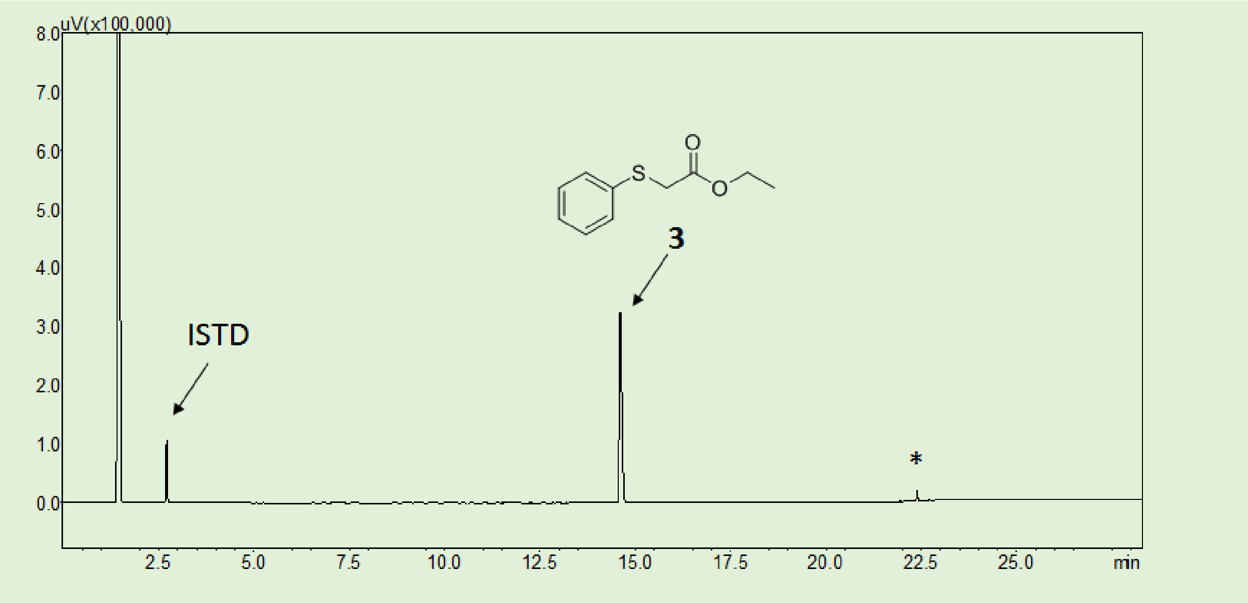

Figure S2:Plot of percentage of conversion over time for Mb-catalyzed formation of α-(phenylthio)acetate (**3**) from thiophenol and EDA. Conversion was determined by gas chromatography using calibration curves with isolated **3**.Reaction conditions: 20 μM Mb, 10mM thiophenol, 20 mM EDA, 10 mM dithionite in oxygen-free phosphate buffer (pH 8.0).

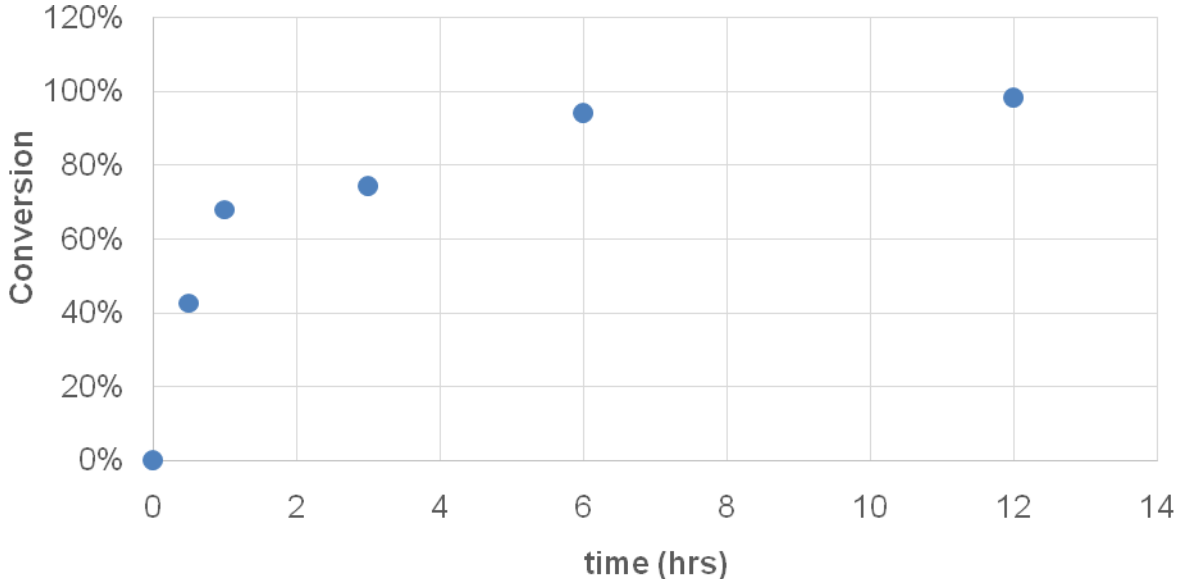

Figure S3: Representative chiral GC chromatograms corresponding to product **21** (a) as authentic racemic standard synthesized using $Rh_2(OAc)_4$catalyst,(b) as produced from the reaction with Mb(F43V) (Entry 3, **Table 3**), (c) as produced from the reaction with Mb(F43V) under optimized conditions (Entry 8, **Table 3**). The two enantiomers of **21** are labeled **ent-A** and **ent-B**.

(a)

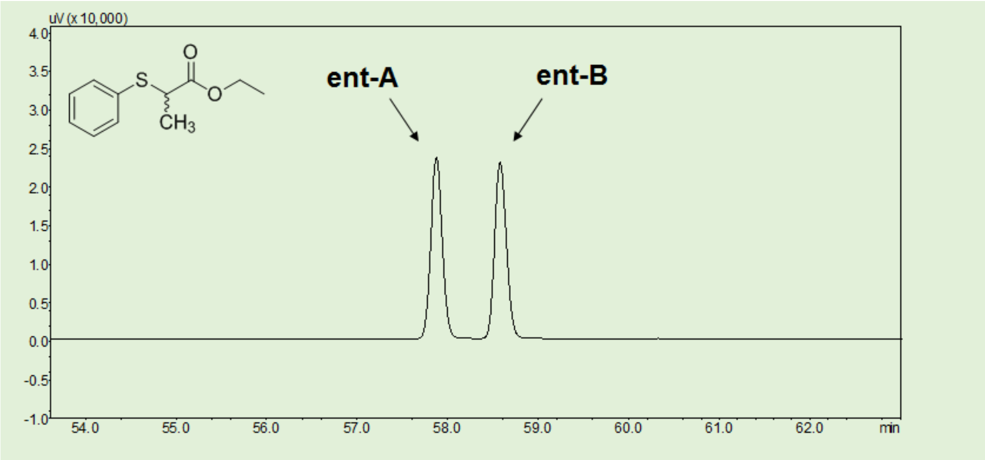

Product	t_R	Peak Area
ent-A	57.8	205549
ent-B	58.6	205466

(b)

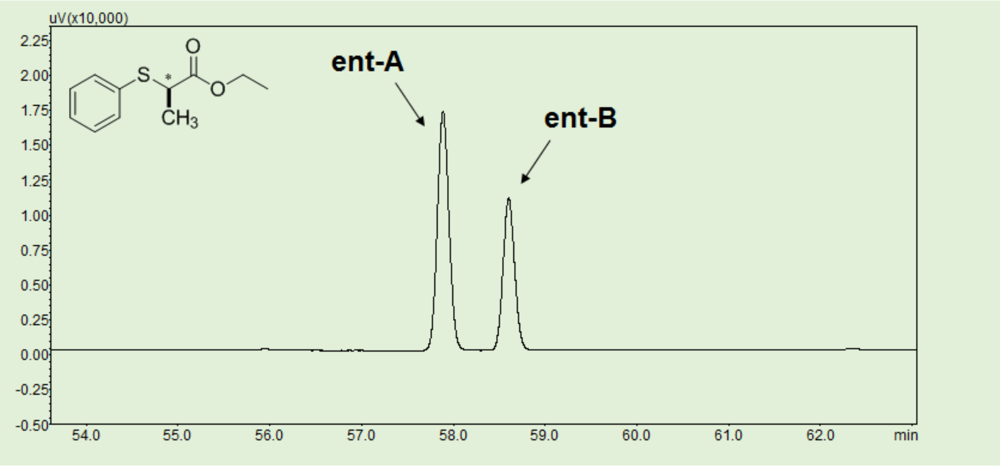

Product	t_R	Peak Area
ent-A	57.8	150365
ent-B	58.6	95242

(c)

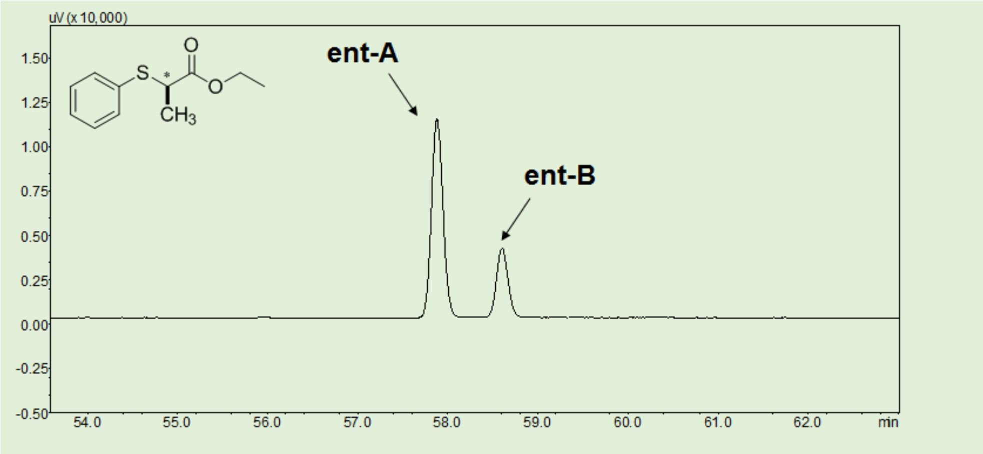

Product	t_R	Peak Area
ent-A	57.8	99768
ent-B	58.6	34578

Figure S4: Hammett plot for the Mb(L29A,H64V)-catalyzed S−H insertion of *para*-substituted thiophenol (*p*-XC_6H_4SH) with EDA. The para substituent (—X) is indicated. Reaction conditions: 5 μM Mb(L29A,H64V), 2.5 mM *p*-XC_6H_4SH, 2.5 mM thiophenol, 1.25 mM EDA, 5 mM dithionite in 80:20 KPi buffer (50mM, pH: 8.0) : MeOH mixture. Reaction time: 20 min.

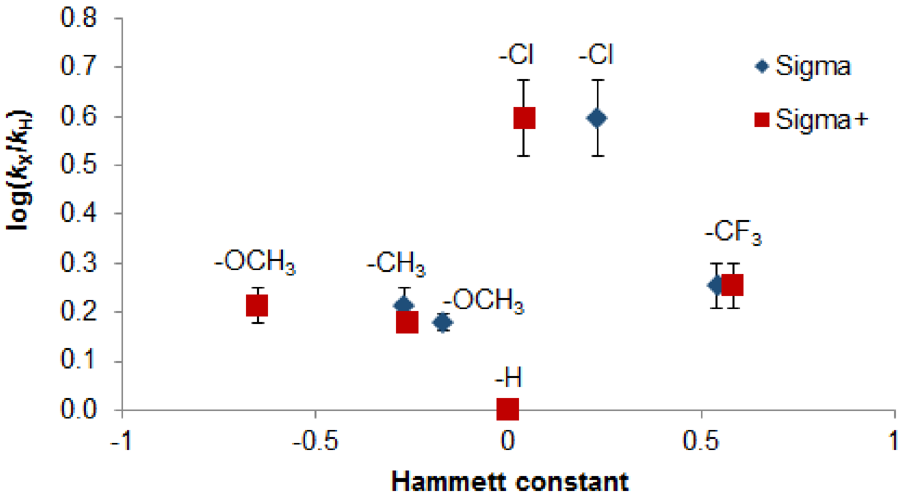

Experimental Procedures

Reagents and Analytical Methods. All the chemicals and reagents were purchased from commercial suppliers (Sigma-Aldrich, Alfa Aesar) and used without any further purification, unless otherwise stated. All dry reactions were carried out under argon atmosphere in oven-dried glassware with magnetic stirring using standard gas-tight syringes, cannulae and septa. ^{1}H and ^{13}C NMR spectra were measured on Bruker DPX-400 (operating at 400 MHz for ^{1}H and 100 MHz for ^{13}C) or Bruker DPX-500 (operating at 500 MHz for ^{1}H and 125 MHz for ^{13}C). Tetramethylsilane (TMS) served as the internal standard (0 ppm) for ^{1}H NMR and $CDCl_3$ was used as the internal standard (77.0 ppm) for ^{13}C NMR. Silica gel chromatography purifications were carried out using AMD Silica Gel 60 230-400 mesh. Gas chromatography (GC) analyses were carried out using a Shimadzu GC-2010 gas chromatograph equipped with a FID detector and aChiral Cyclosil-B column (30 m x 0.25 mm x 0.25 μm film). Separation methodfor calculation of TON and TTN values: 1 μL injection, injector temp.: 200 ºC, detector temp: 300 ºC. Gradient: column temperature set at 140 ºC for 3 min, then to 160 ºC at 1.8 ºC/min, then to 165 ºC at 1 ºC/min, then to 245 ºC at 25 ºC/min. Total run time was 28.31 min. Enantiomeric excess for product **21** was determined using the following separation method: 1 μL injection, injector temp.: 200 ºC, detector temp: 300 ºC. Gradient: column temperature set at 80 ºC for 3 min, then to 180 ºC at 1.00 ºC/min, then to 200 ºC at 2 ºC/min, then to 245 ºC at 25 ºC/min. Total run time was 120.80 min.

Protein expression and purification. Wild-type Mb and the engineered Mb variants were expressed in *E. coli* BL21(DE3) cells as described previously.[1]Briefly, cells were grown in TB medium (ampicillin, 100 mg L^{-1}) at 37 °C (150 rpm) until OD_{600} reached 0.6. Cells were then

induced with 0.25 mM β-D-1-thiogalactopyranoside (IPTG) and 0.3 mM δ-aminolevulinic acid (ALA). After induction, cultures were shaken at 150 rpm and 27 °C and harvested after 20 h by centrifugation at 4000 rpm at 4 °C. After cell lysis by sonication, the proteins were purified by Ni-affinity chromatography using the following buffers: loading buffer (50 mM Kpi, 800 mM NaCl, pH 7.0), wash buffer 1 (50 mM Kpi, 800 mM NaCl, pH 6.2), wash buffer 2 (50 mM Kpi, 800 mM NaCl, 250 mM glycine, pH 7.0) and elution buffer (50 mM Kpi, 800 mM NaCl, 300 mM L-histidine, pH 7.0). After buffer exchange (50 mM Kpi, pH 7.0), the proteins were stored at +4 °C. Myoglobin concentration was determined using an extinction coefficient ε_{410} = 157 mM^{-1} cm^{-1}.[2]

S-H insertion reactions. Initial reactions (**Table 1**) were carried out at a 400 µL scale using 20 µM myoglobin, 10 mM thiophenol, 5 mM EDA, and 10 mM sodium dithionite. In a typical procedure, a solution containing sodium dithionate (100 mM stock solution) in potassium phosphate buffer (50 mM, pH 8.0) was degassed by bubbling argon into the mixture for 4 min in a sealed vial. A buffered solution containing myoglobin was carefully degassed in a similar manner in a separate vial. The two solutions were then mixed together via cannula. Reactions were initiated by addition of 10 µL of thiophenol (from a 0.4 M stock solution in methanol), followed by the addition of 10µL of EDA (from a 0.2 M stock solution in methanol) with a syringe, and the reaction mixture was stirred for 12 h at room temperature, under positive argon pressure. For the optimization of the thiophenol:EDA ratio, reactions were performed according to the general procedure described above, using 20 µM of protein, 10 mM of thiophenol and variable amounts of EDA (2.5 mM EDA to 40mM EDA). Optimization of the substrate loading was done in a similar manner, using 20 µM Mb, variable quantities of thiophenol (from 10 to

80mM final concentration), and variable quantities of EDA (from 20 to 160 mM final concentration), maintaining an thiophenol:EDA ratio of 1:2 at all times. Enzyme concentration optimization was carried according to the general procedure along with varying the enzyme concentration from 20 µM to 1 µM of Mb(L29A, H64V)and 10 mM thiophenol (10 µL of 0.4 M stock solution in methanol), and 20 mM EDA (10 µL of 0.8 M stock solution in methanol). Reactions for TTN determination were carried out according to the general procedure described above using 2.5 µM Mb(L29A, H64V), 10 mM thiophenol (10 µL of 0.4 M stock solution in methanol), and 20 mM EDA (10 µL of 0.8 M stock solution in methanol).

Preparative-scale reaction. A solution containing sodium dithionate (100 mM stock solution, 1 mL, 10 mM) in potassium phosphate buffer (50 mM, pH 8.0, 5.87 mL) and 466 µL of MeOH(>5% of reaction volume) was degassed by bubblingargon into the mixture for 20 min in a sealed vial. A buffered solution containing 20 µM Mb(L29A, H64V) (2.63 mL of 76 µM stock solution) was carefully degassed in a similarmanner in a separate vial. The two solutions were then mixed together via cannula. Reactionswere initiated by addition of 10.3 µL of pure thiophenol, followed by the addition of 24 µL of pure EDAwith a syringe, and the reaction mixture was stirred for 12 h at room temperature, under positiveargon pressure. The reaction mixture was extracted with dichloromethane (4 x 10 mL), organiclayer evaporated under reduced pressure and the residue was purified by flash columnchromatography (10% ethyl acetate in hexanes) to yield product 3 as colorlessliquid (13.2 mg, 67%).

Product analysis: The reactions were analyzed by adding 20 µL of internal standard (benzodioxole, 50 mM in methanol) to the reaction mixture, followed by extraction with 400 µL

of dichloromethane and separated organic layer was analyzed by GC-FID (see **Reagents and Analytical Methods** section for details on GC analyses). Calibration curves forquantification of the different S-H insertion products were constructed using authentic standards prepared synthetically using $Rh_2(OAc)_4$ as the catalyst as described in **Synthetic Procedures**. All measurements were performed at least in duplicate. For each experiment, negative control samples containing either no enzyme or no reductant were included.

Synthetic Procedures:

General procedure for Rh-catalyzed authentic S-H insertion products:

To a flame dried round bottom flask under argon, equipped with a stir bar was added thiol (1 equiv.) and $Rh_2(OAC)_4$ (5 mol%) in dichloromethane (2-3 mL). To this solution was added a solution of diazo compound (1 equiv.) in dichloromethane (1-2 mL) by slow addition over 30-45 minutes at 0°C.The resulting mixture was stirred at room temperature for another 2-3 hour. The solvent was removed under vacuum and the crude mixture was purified by 9:1 hexanes to diethyl ether using flash chromatography to obtained S-H insertion products in good to excellent yield. The identity of the S-H insertion productswas determined using GC-MS, ^{1}H and ^{13}CNMR.

Ethyl 2-(phenylthio)acetate (3)

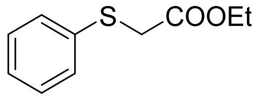

Following the standard procedure, % yield (86), GC-MS m/z (% relative intensity): 196(57.3), 123(100), 109(12.0), 77(10.6);^{1}H NMR ($CDCl_3$, 500 MHz): δ 7.32 (d, J = 7.5 Hz, 2H), 7.26-7.21 (m, 3H), 4.18 (q, J = 7.0 Hz, 2H), 3.63 (s, 2H), 1.24 (t, J = 7.0 Hz, 3H) ppm;^{13}C NMR ($CDCl_3$, 125 MHz): δ 169.7, 135.0, 130.0, 129.0, 126.9, 61.5, 36.7, 14.1 ppm.

Ethyl 2-(*p*-tolylthio)acetate (11)

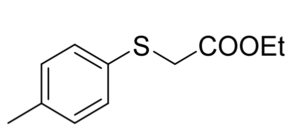

Following the standard procedure, % yield (79), GC-MS m/z (% relative intensity): 210(67.0), 137(100), 99(17.9);^{1}H NMR ($CDCl_3$, 500 MHz): δ 7.34 (d, J = 8.5 Hz, 2H), 7.12 (d, J = 8.0 Hz,

2H), 4.17 (q, J = 7.0 Hz, 2H), 3.57 (s, 2H), 2.32 (s, 3H), 1.23 (t, J = 7.0 Hz, 3H) ppm, ^{13}C NMR ($CDCl_3$, 125 MHz): δ 169.8, 137.3, 130.9, 129.8, 61.4, 37.4, 21.1, 14.1 ppm.

Ethyl 2-((4-methoxyphenyl)thio)acetate (12)

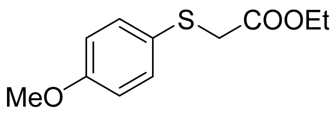

Following the standard procedure, % yield (83), GC-MS m/z (% relative intensity): 226(100), 153(88.0), 139(43.8), 109(18.5);^{1}H NMR ($CDCl_3$, 500 MHz): δ 7.41 (d, J = 9.0 Hz, 2H), 6.83 (d, J = 9.0 Hz, 2H), 4.14 (q, J = 7.5 Hz, 2H), 3.77 (s, 3H), 3.49 (s, 2H), 1.21 (t, J = 7.5 Hz, 3H) ppm,^{13}C NMR ($CDCl_3$, 125 MHz): δ 169.9, 159.6, 134.2, 124.9, 114.6, 61.3, 55.3, 38.6, 14.1 ppm.

Ethyl 2-((4-chlorophenyl)thio)acetate (13)

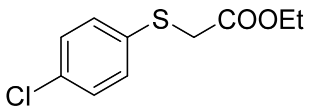

Following the standard procedure, % yield (85), GC-MS m/z (% relative intensity): 230(65.5), 157(100), 143(8.4), 108(9.2);^{1}H NMR ($CDCl_3$, 500 MHz): δ 7.35 (d, J = 8.5 Hz, 2H), 7.26 (d, J = 8.5 Hz, 2H), 4.17 (q, J = 7.0 Hz, 2H), 3.59 (s, 2H), 1.23 (t, J = 7.0 Hz, 3H) ppm, ^{13}C NMR ($CDCl_3$, 125 MHz): δ 169.4, 133.5, 133.2, 131.5, 129.2, 61.6, 36.8, 14.1 ppm.

Ethyl 2-((4-bromophenyl)thio)acetate (14)

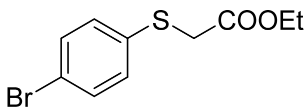

Following the standard procedure, % yield (79), GC-MS m/z (% relative intensity):274(100), 202(45.7), 201(47.3), 122(74.0), 108(17.4); ^{1}H NMR ($CDCl_3$, 400 MHz): δ 7.39 (d, J = 7.6 Hz, 2H), 7.26 (d, J = 7.2 Hz, 2H), 4.16 (q, J = 7.2 Hz, 2H), 3.58 (s, 2H), 1.22 (t, J = 7.2 Hz, 3H) ppm, ^{13}C NMR ($CDCl_3$, 125 MHz): δ169.3, 134.2, 132.1, 131.5, 120.9, 61.6, 36.6, 14.1 ppm.

Ethyl 2-((4-(trifluoromethyl)phenyl)thio)acetate (15)

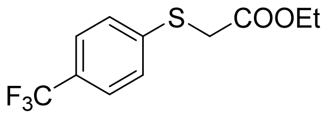

Following the standard procedure, % yield (72), GC-MS m/z (% relative intensity): 264(100), 191(98.1), 171(33.6);^{1}H NMR ($CDCl_3$, 500 MHz): δ7.54 (d, J = 8.0 Hz, 2H), 7.45 (d, J = 8.0 Hz, 2H), 4.21 (q, J = 7.0 Hz, 2H), 3.70 (s, 2H), 1.25 (t, J = 7.0 Hz, 3H) ppm, ^{13}C NMR ($CDCl_3$, 125 MHz): δ 169.1, 140.5, 128.7, 128.5, 128.2, 128.1,127.3, 125.8, 125.7, 125.1, 122.9, 120.7 61.8, 35.3, 14.0 ppm.

Ethyl 2-(m-tolylthio)acetate (16)

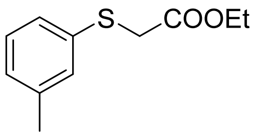

Following the standard procedure, % yield (81), GC-MS m/z (% relative intensity): 210(67.5), 137(100), 91(17.9);^{1}H NMR ($CDCl_3$, 400 MHz): δ 7.22-7.17 (m, 3H), 7.03 (d, J = 6.4 Hz, 1H),

4.18 (q, J = 6.8 Hz, 2H), 3.61 (s, 2H), 2.31 (s, 3H), 1.23 (t, J = 7.2 Hz, 3H) ppm, ^{13}C NMR ($CDCl_3$, 100 MHz): δ 169.7, 138.8, 134.7, 130.5, 128.8, 127.8, 126.9, 61.5, 36.7, 21.3, 14.1 ppm.

Ethyl 2-(o-tolylthio)acetate (17)

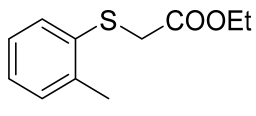

Following the standard procedure, % yield (84), GC-MS m/z (% relative intensity): 210(74.7), 164(35.3), 137(100), 91(31.8);^{1}H NMR ($CDCl_3$, 400 MHz): δ 7.35 (d, J = 6.4 Hz, 1H), 7.16-7.14 (m, 3H), 4.17 (q, J = 7.2 Hz, 2H), 3.61 (s, 2H), 2.41 (s, 3H), 1.23 (t, J = 7.2 Hz, 3H) ppm, ^{13}C NMR ($CDCl_3$, 100 MHz): δ169.6, 138.2, 134.1, 130.2, 129.4, 126.8, 126.6, 61.5, 35.9, 20.3, 14.1 ppm.

***tert*-Butyl 2-(phenylthio)acetate (18)**

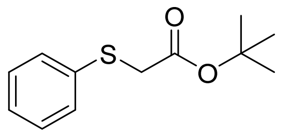

Following the standard procedure, % yield (86), GC-MS m/z (% relative intensity): 224(16.5), 168(33.1), 123(58.6), 57(100);^{1}H NMR ($CDCl_3$, 500 MHz): δ 7.41 (d, J = 7.5 Hz, 2H), 7.29-7.27 (m, 2H), 7.22 (d, J = 5.6 Hz, 1H), 3.55 (s, 2H), 1.39 (s, 9H) ppm, ^{13}C NMR ($CDCl_3$, 125 MHz): δ 168.8, 135.3, 129.8, 128.9, 126.7, 81.9, 37.7, 27.9 ppm.

Cyclohexyl 2-(phenylthio)acetate (19)

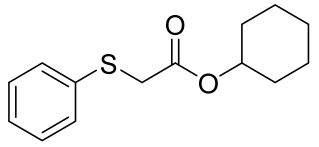

Following the standard procedure, % yield (78), GC-MS m/z (% relative intensity): 250(55.9), 168(28.7), 123(65.8), 83(100), 55(66.9);^{1}H NMR ($CDCl_3$, 500 MHz): δ 7.42 (d, *J* = 6.0 Hz, 2H), 7.30-7.20 (m, 3H), 4.79-4.76 (m, 1H), 3.62 (s, 2H), 1.79-1.23 (m, 10H) ppm, ^{13}C NMR ($CDCl_3$, 125 MHz): δ 169.2, 135.1, 129.9, 128.9, 126.8, 73.9, 36.9, 31.4, 25.3, 23.5 ppm.

Benzyl 2-(phenylthio)acetate (20)

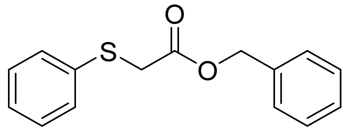

Following the standard procedure, % yield (86), GC-MS m/z (% relative intensity): 258(69.8), 123(61.1), 91(100), 65(11.5);^{1}H NMR ($CDCl_3$, 400 MHz): δ 7.34-7.21 (m, 10H), 5.14 (s, 2H), 3.68 (s, 2H) ppm, ^{13}C NMR ($CDCl_3$, 100 MHz): δ 169.6, 135.3, 134.8, 130.1, 129.1, 128.6, 128.4, 128.3, 127.0, 67.3, 36.7 ppm.

Ethyl 2-(phenylthio)propanoate (21)

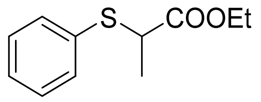

Following the standard procedure, % yield (62), GC-MS m/z (% relative intensity): 210(41.9), 137(100), 109(24.1);^{1}H NMR ($CDCl_3$, 400 MHz): δ7.46 (d, *J* = 6.8 Hz, 2H) 7.31-7.28 (m, 3H),

4.13 (q, J = 7.2 Hz, 2H), 3.80 (q, J = 7.2 Hz, 1H), 1.48 (d, J = 7.2 Hz, 3H), 1.18 (t, J = 7.2 Hz, 3H) ppm, ^{13}C NMR ($CDCl_3$, 100 MHz): δ 172.7, 133.0, 128.9, 127.9, 61.2, 45.2, 17.3, 14.0 ppm.

Ethyl 2-(benzylthio)acetate (28)

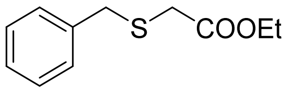

Following the standard procedure, % yield (81), GC-MS m/z (% relative intensity): 210(23.4), 123(86.8), 91(100), 65(11.5);^{1}H NMR ($CDCl_3$, 500 MHz): δ 7.33-7.30 (m, 4H), 7.27-7.24 (m, 1H), 4.20 (q, J = 7.0 Hz, 2H), 3.83 (s, 2H), 3.07 (s, 2H), 1.31 (t, J = 7.0 Hz, 3H) ppm, ^{13}C NMR ($CDCl_3$, 125 MHz): δ 170.4, 137.2, 129.2, 128.5, 127.2, 61.3, 36.3, 32.3, 14.2 ppm.

Ethyl 2-((4-methylbenzyl)thio)acetate (29)

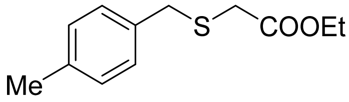

Following the standard procedure, % yield (76), GC-MS m/z (% relative intensity): 224(24.9), 137(90.7), 105(100), 79(11.8);^{1}H NMR ($CDCl_3$, 400 MHz): δ 7.23 (d, J = 7.2 Hz, 2H), 7.13 (d, J = 7.2 Hz, 2H), 4.20 (q, J = 7.2 Hz, 2H), 3.79 (s, 2H), 3.05 (s, 2H), 2.33 (s, 3H), 1.30 (t, J = 7.2 Hz, 3H) ppm, ^{13}C NMR ($CDCl_3$, 100 MHz): δ 170.4, 136.8, 134.1, 129.2, 61.2, 36.0, 32.2, 21.1, 14.2 ppm.

Ethyl 2-((4-methoxybenzyl)thio)acetate (30)

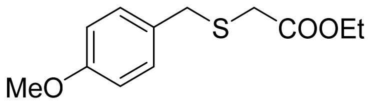

Following the standard procedure, % yield (79), GC-MS m/z (% relative intensity): 240(9.9), 153(9.5), 121(100), 77(4.8); ^{1}H NMR (CDCl$_3$, 500 MHz): δ 7.26 (d, *J* = 8.5 Hz, 2H), 6.86 (d, *J*= 8.5 Hz, 2H), 4.20 (q, *J* = 7.0 Hz, 2H), 3.79 (br s, 5H), 3.05 (s, 2H), 1.31 (t, *J* = 7.0 Hz, 3H) ppm, ^{13}C NMR (CDCl$_3$, 125 MHz): δ 170.5, 158.8, 130.3, 129.2, 113.9, 61.3, 55.3, 35.7, 32.2, 14.2 ppm.

Ethyl 2-((4-chlorobenzyl)thio)acetate (31)

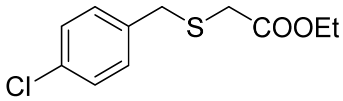

Following the standard procedure, % yield (72), GC-MS m/z (% relative intensity): 244(25.8), 157(100), 76.9(125), 89(16.2); ^{1}H NMR (CDCl$_3$, 400 MHz): δ 7.26 (br s, 4H), 4.18 (q, *J* = 7.2 Hz, 2H), 3.77 (s, 2H), 3.02 (s, 2H), 1.28 (t, *J* = 7.2 Hz, 3H) ppm, ^{13}C NMR (CDCl$_3$, 100 MHz): δ 170.2, 135.8, 133.0, 130.5, 128.6, 61.3, 35.5, 32.1, 14.1 ppm.

Ethyl 2-(cyclohexylthio)acetate (32)

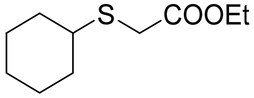

Following the standard procedure, % yield (72), GC-MS m/z (% relative intensity): 202(25.8), 115(100), 81(81.7), 67(26.8), 55(31.5); ^{1}H NMR (CDCl$_3$, 500 MHz): δ 4.18 (q, *J* = 7.0 Hz, 2H),

3.22 (s, 2H), 2.79-2.76 (m, 1H), 1.97-1.96 (m, 2H), 1.75 (m, 2H), 1.60-1.58 (m, 1H), 1.33-1.20 (m, 8H) ppm, ^{13}C NMR ($CDCl_3$, 125 MHz): δ170.9, 61.2, 43.9, 33.1, 32.1, 25.9, 25.7, 14.1 ppm.

Ethyl 2-(octylthio)acetate (33)

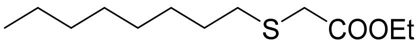

Following the standard procedure, % yield (67), GC-MS m/z (% relative intensity): 232(33.5), 159(15.6), 145(100), 88(89.7), 69(80.7), 55(21.5);^{1}H NMR ($CDCl_3$, 400 MHz): δ 4.13-4.11 (m, 2H), 3.14 (s, 2H), 2.58-2.55 (m, 2H), 1.55-1.52 (m, 2H), 1.32-1.22 (m, 13H), 0.82 (m, 3H) ppm, ^{13}C NMR ($CDCl_3$, 100 MHz): δ 170.5, 61.1, 33.6, 32.6, 31.7, 29.1, 28.9, 28.7, 22.5, 14.1, 13.9 ppm.

Benzyl 2-(benzylthio)acetate (34)

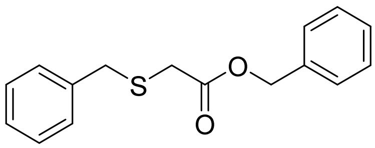

Following the standard procedure, % yield (82), GC-MS m/z (% relative intensity): 272(1.6), 181(83.6), 107(16.8), 91(100), 65(8.2);^{1}H NMR ($CDCl_3$, 400 MHz): δ 7.39-7.26 (m, 10H), 5.18 (s, 2H), 3.82 (s, 2H), 3.13 (s, 2H) ppm, ^{13}C NMR ($CDCl_3$, 100 MHz): δ 170.2, 137.1, 135.6, 129.2, 128.6, 128.5, 128.4, 128.3, 127.3, 67.0, 36.3, 32.3 ppm.

Benzyl 2-(cyclohexylthio)acetate (35)

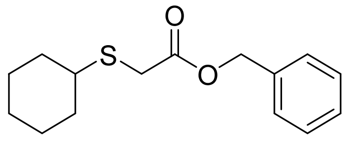

Following the standard procedure, % yield (76), GC-MS m/z (% relative intensity): 264(17.3), 173(25.3), 115(61.1), 91(100), 81(58.4), 55(25.8);^{1}H NMR ($CDCl_3$, 400 MHz): δ 7.35-7.33 (m, 5H), 5.16 (s, 2H), 3.27 (s, 2H), 2.74 (m, 1H), 1.95-1.93 (m, 2H), 1.72 (m, 2H), 1.59 (br s, 1H), 1.29-1.21 (m, 5H) ppm, ^{13}C NMR ($CDCl_3$, 100 MHz): δ 170.6, 135.7, 128.5, 128.3, 66.9, 43.9, 33.1, 32.0, 25.9, 25.7 ppm.

Ethyl 2-(phenylthio)pent-4-enoate (38)

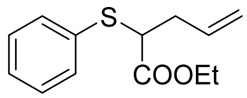

To a flame dried round bottom flask under argon, equipped with a stir bar was added allyl phenyl sulfide(1 equiv.) and $Rh_2(OAC)_4$ (5 mol%) in dichloromethane (2-3 mL). To this solution was added a solution of EDA (1 equiv.) in dichloromethane (1-2 mL) over 30 minutes at 0°C. The resulting mixture was stirred at room temperature for overnight. The solvent was removed under vacuum and the crude mixture was purified by 9:1 hexanes to diethyl ether using flash chromatography to obtained [2,3]-sigmatropic rearrangement product in good yield.

% yield (80), GC-MS m/z (% relative intensity): 236(61.9), 195(81.5), 163(88.1), 149(98.1), 121 (93.7), 109 (100);^{1}H NMR ($CDCl_3$, 500 MHz): δ 7.47 (d, J = 5.5 Hz, 2H), 7.30-7.26 (m, 3H), 5.85-5.76 (m, 1H), 5.15-5.08 (m, 2H), 4.18-4.09 (m, 2H), 3.71-3.67 (m, 1H), 2.66-2.60 (m, 1H),

2.54-2.49 (m, 1H), 1.18-1.14 (m, 3H) ppm, ^{13}C NMR ($CDCl_3$, 125 MHz): δ 171.6, 133.9, 133.1, 128.9, 128.0, 118.0, 61.1, 50.3, 35.8, 14.1 ppm.

References

[1] Bordeaux, M.; Tyagi, V.; Fasan, R., *Angew. Chem. Int. Ed.,* 2014, in press.

[2] Redaelli, C.; Monzani, E.; Santagostini, L.; Casella, L.; Sanangelantoni, A. M.; Pierattelli, R.; Banci, L., *Chembiochem,* 2002, **3**, 226.

^{1}H and ^{13}C NMR spectra:

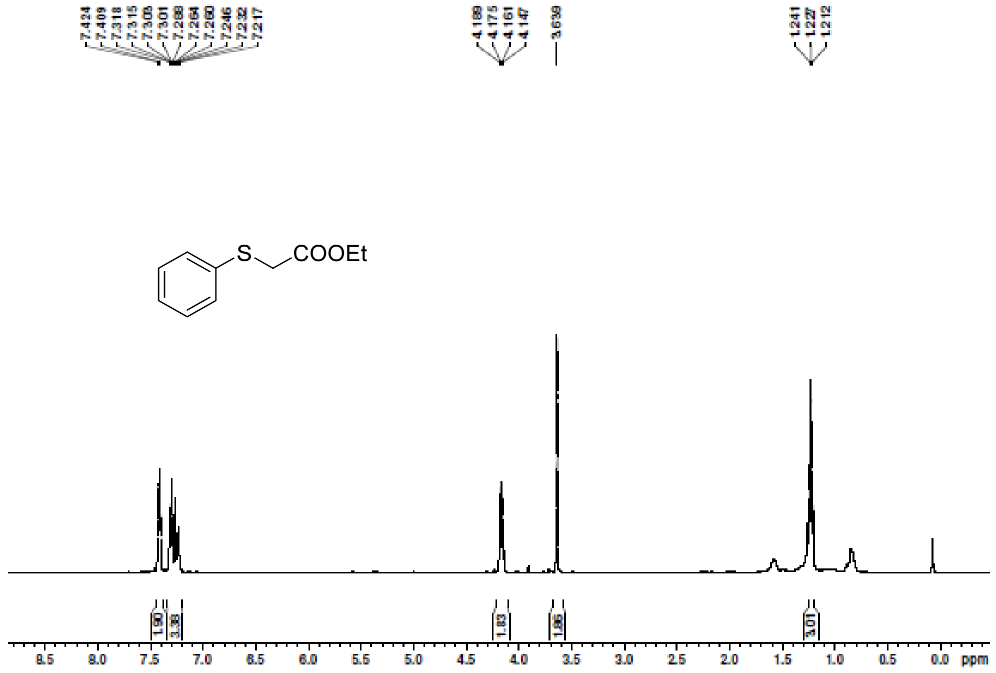

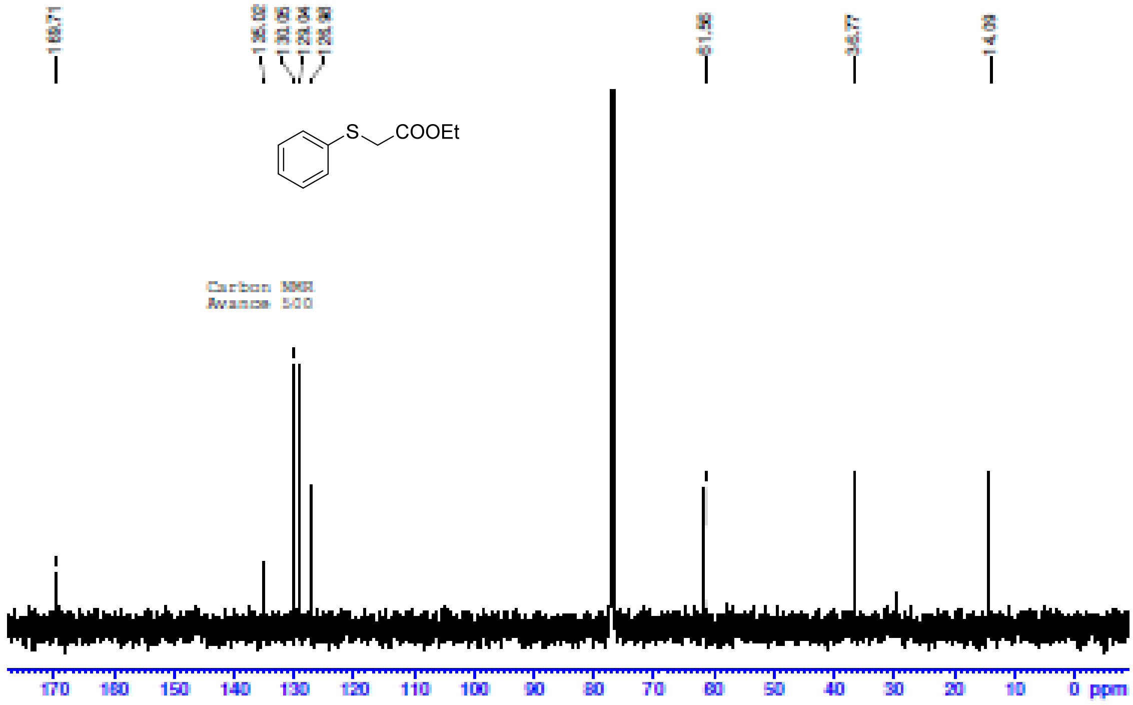

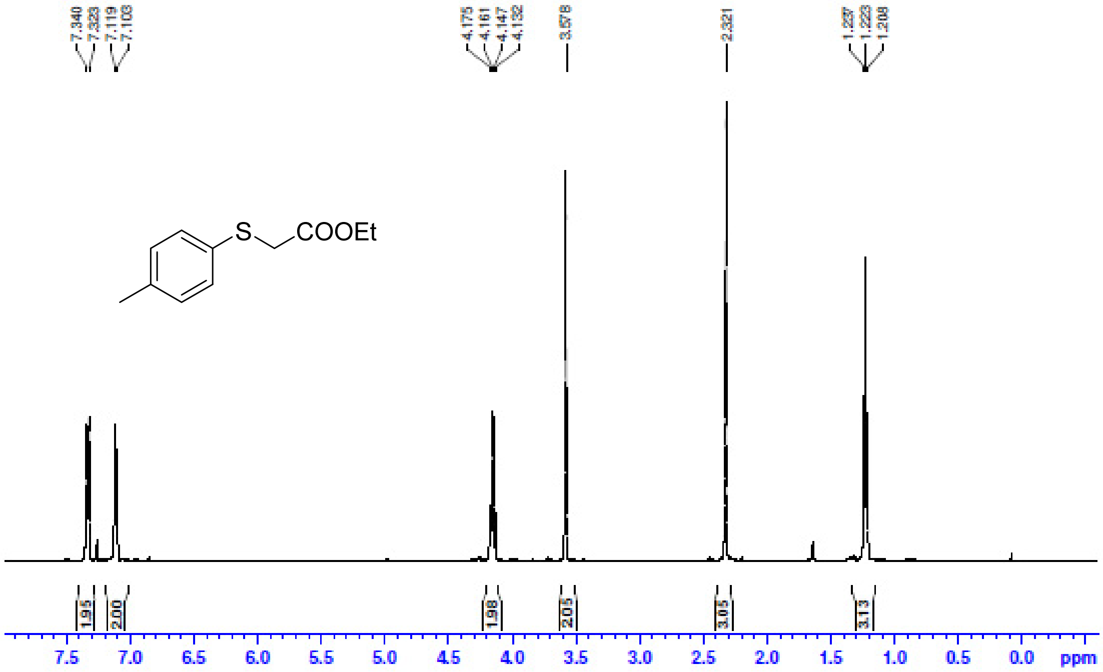
7.340
7.323
7.119
7.103
4.175
4.161
4.147
4.132
3.578
2.321
1.237
1.223
1.208
S
COOEt
1.95
2.00
1.98
2.05
3.05
3.13
7.5 7.0 6.5 6.0 5.5 5.0 4.5 4.0 3.5 3.0 2.5 2.0 1.5 1.0 0.5 0.0 ppm

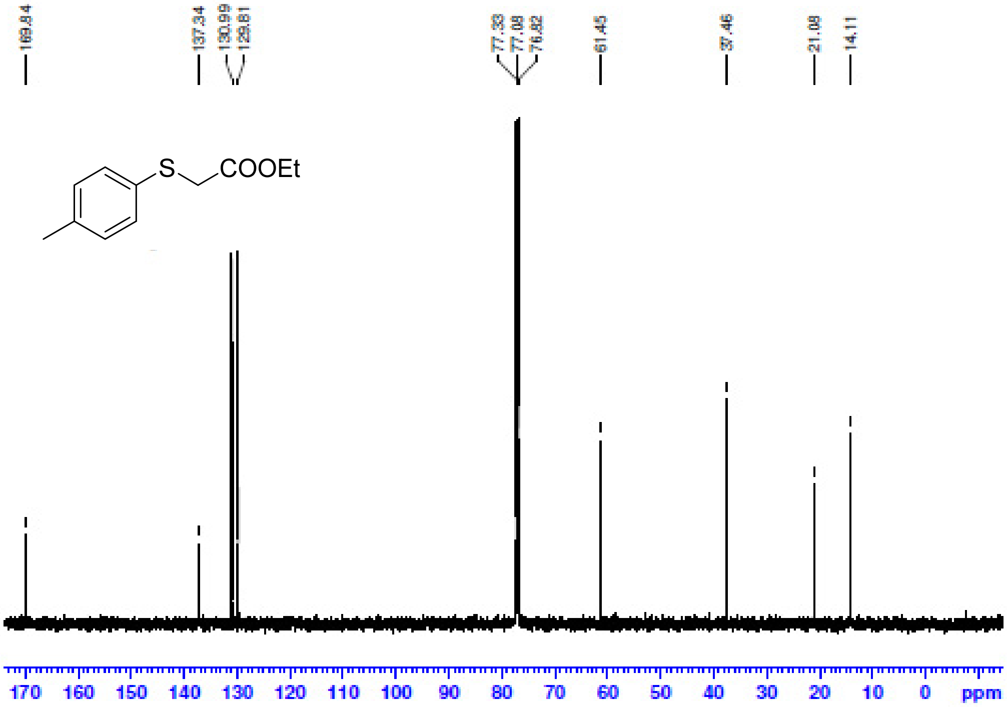
169.84
137.34
130.99
129.81
77.33
77.08
76.82
61.45
37.46
21.08
14.11
S
COOEt
170 160 150 140 130 120 110 100 90 80 70 60 50 40 30 20 10 0 ppm

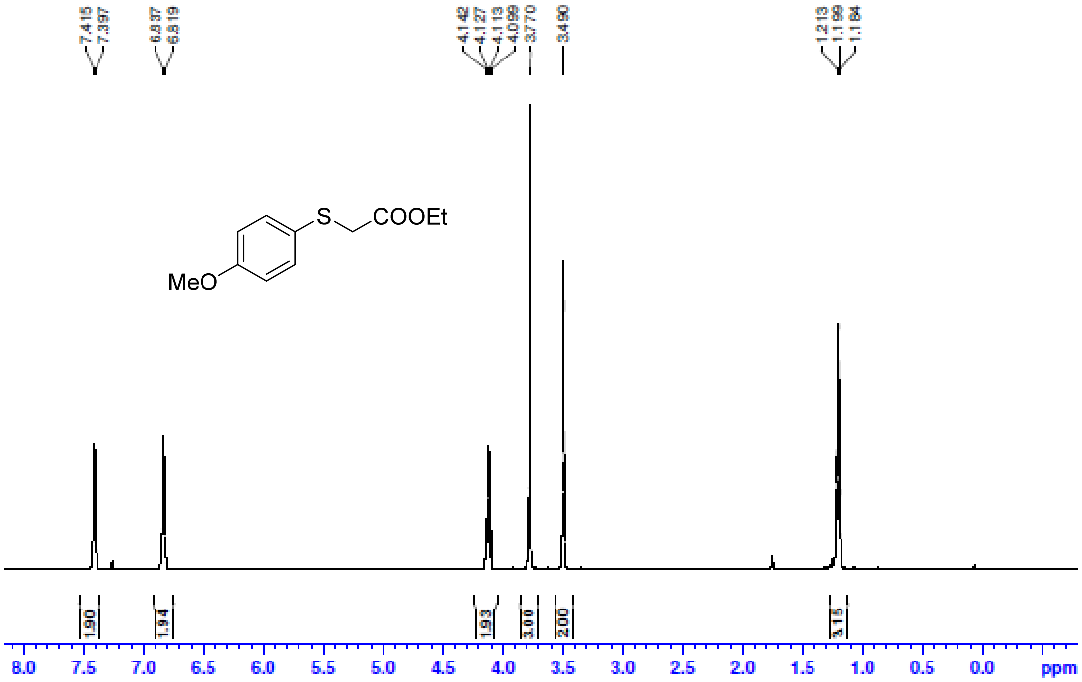
7.415
7.397
6.837
6.819
4.142
4.127
4.113
4.099
3.770
3.490
1.213
1.199
1.184
S
COOEt
MeO
1.90
1.94
1.93
3.00
2.00
3.15
8.0 7.5 7.0 6.5 6.0 5.5 5.0 4.5 4.0 3.5 3.0 2.5 2.0 1.5 1.0 0.5 0.0 ppm

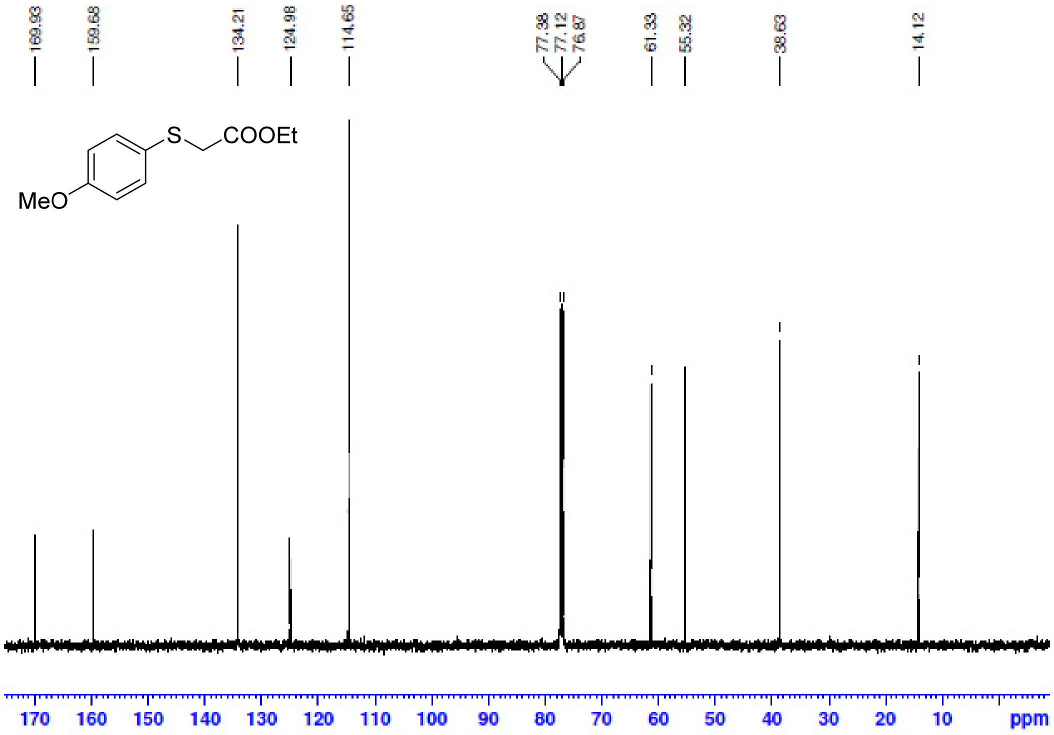
169.93
159.68
134.21
124.98
114.65
77.38
77.12
76.87
61.33
55.32
38.63
14.12
S
COOEt
MeO
170 160 150 140 130 120 110 100 90 80 70 60 50 40 30 20 10 ppm

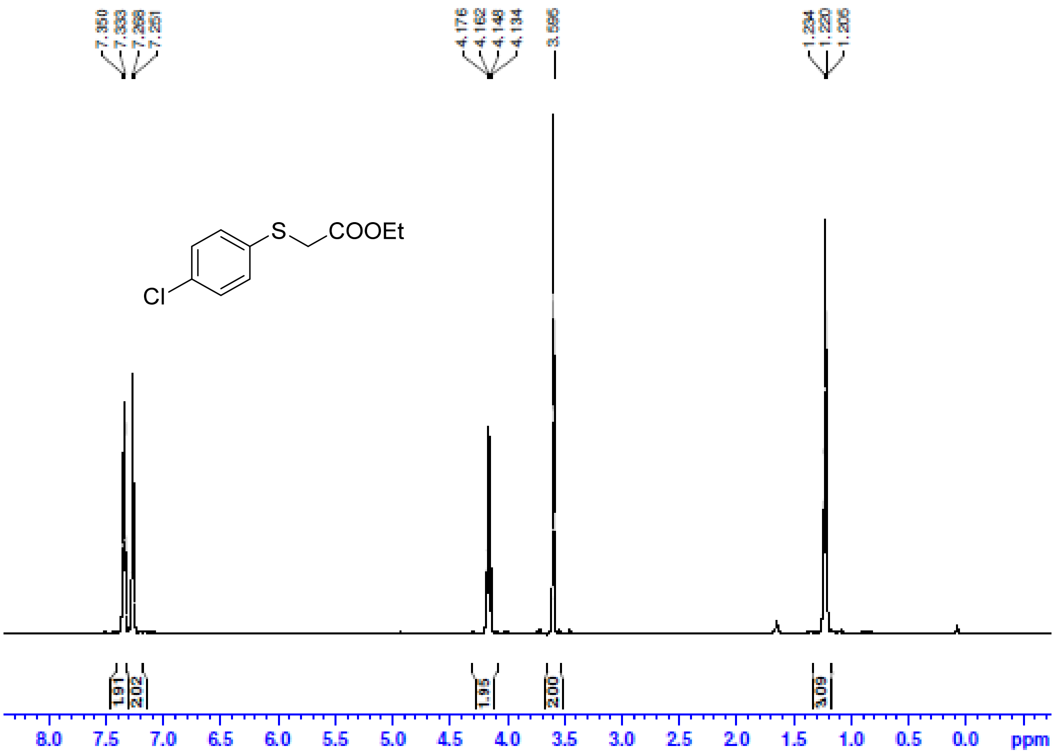
7.350
7.333
7.268
7.251
4.176
4.162
4.148
4.134
3.595
1.234
1.220
1.206
S
COOEt
Cl
1.91
2.02
1.95
2.00
3.09
8.0 7.5 7.0 6.5 6.0 5.5 5.0 4.5 4.0 3.5 3.0 2.5 2.0 1.5 1.0 0.5 0.0 ppm

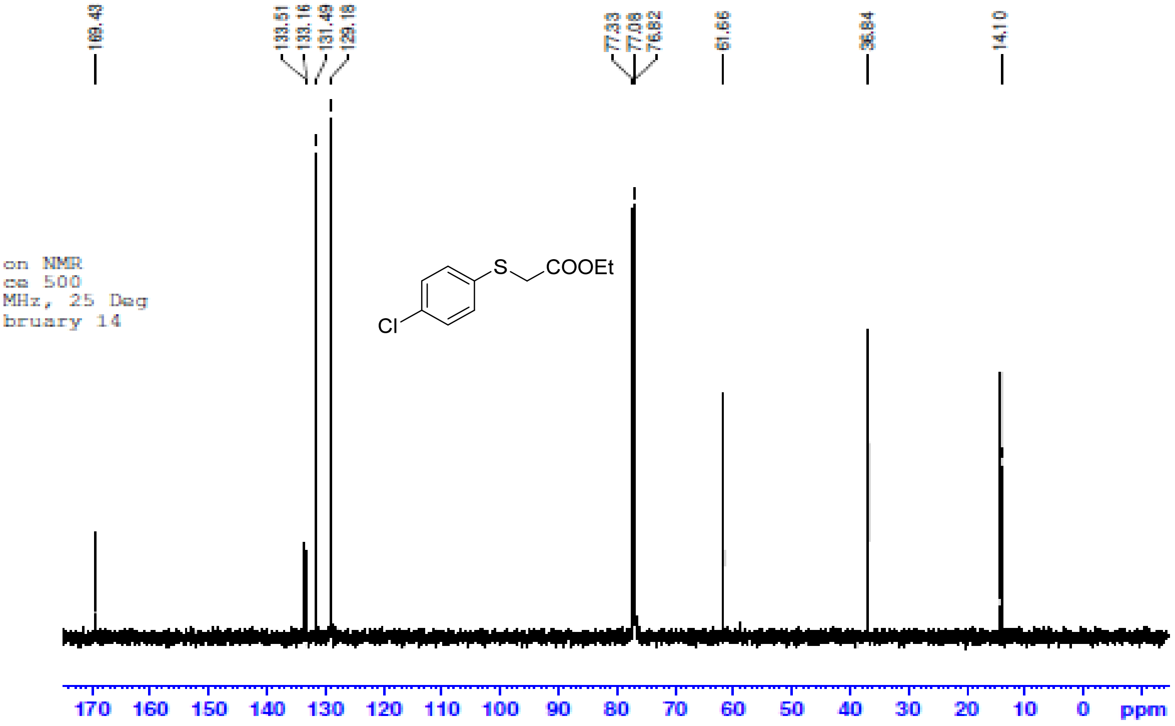
169.43
133.51
133.16
131.49
129.18
77.33
77.08
76.82
61.66
36.84
14.10
on NMR
ce 500
MHz, 25 Deg
bruary 14
S
COOEt
Cl
170 160 150 140 130 120 110 100 90 80 70 60 50 40 30 20 10 0 ppm

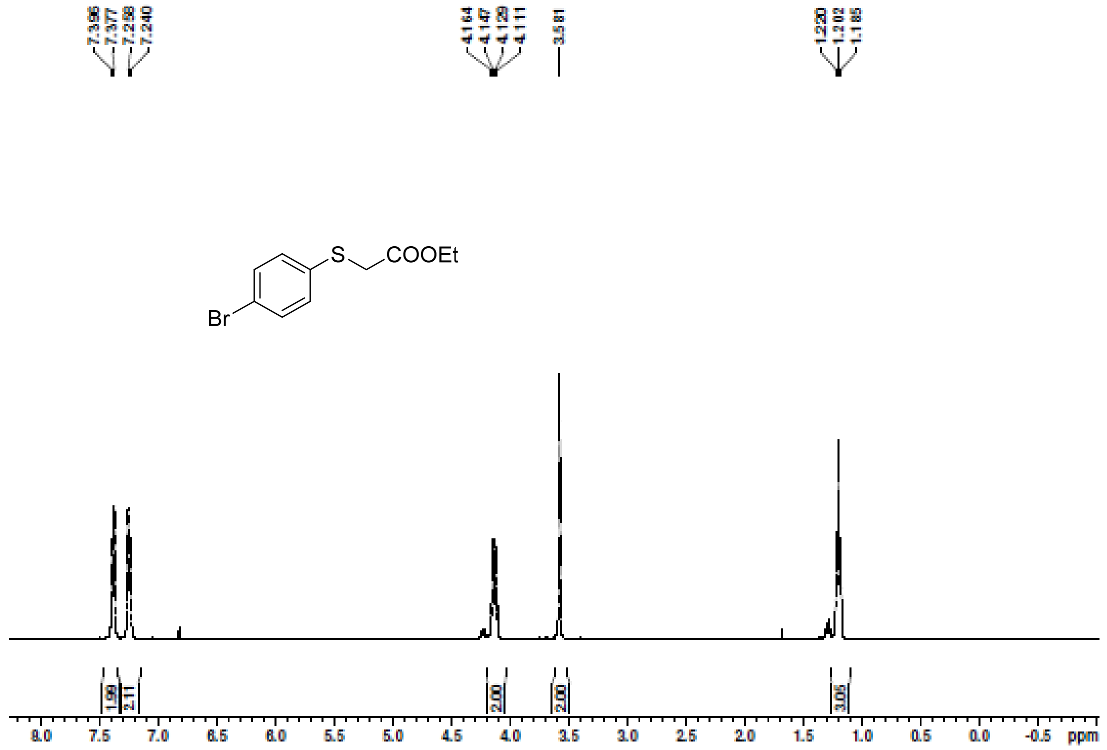
7.396
7.377
7.258
7.240
4.164
4.147
4.129
4.111
3.581
1.220
1.202
1.185
S
COOEt
Br
1.99
2.11
2.00
2.00
3.05
8.0 7.5 7.0 6.5 6.0 5.5 5.0 4.5 4.0 3.5 3.0 2.5 2.0 1.5 1.0 0.5 0.0 -0.5 ppm

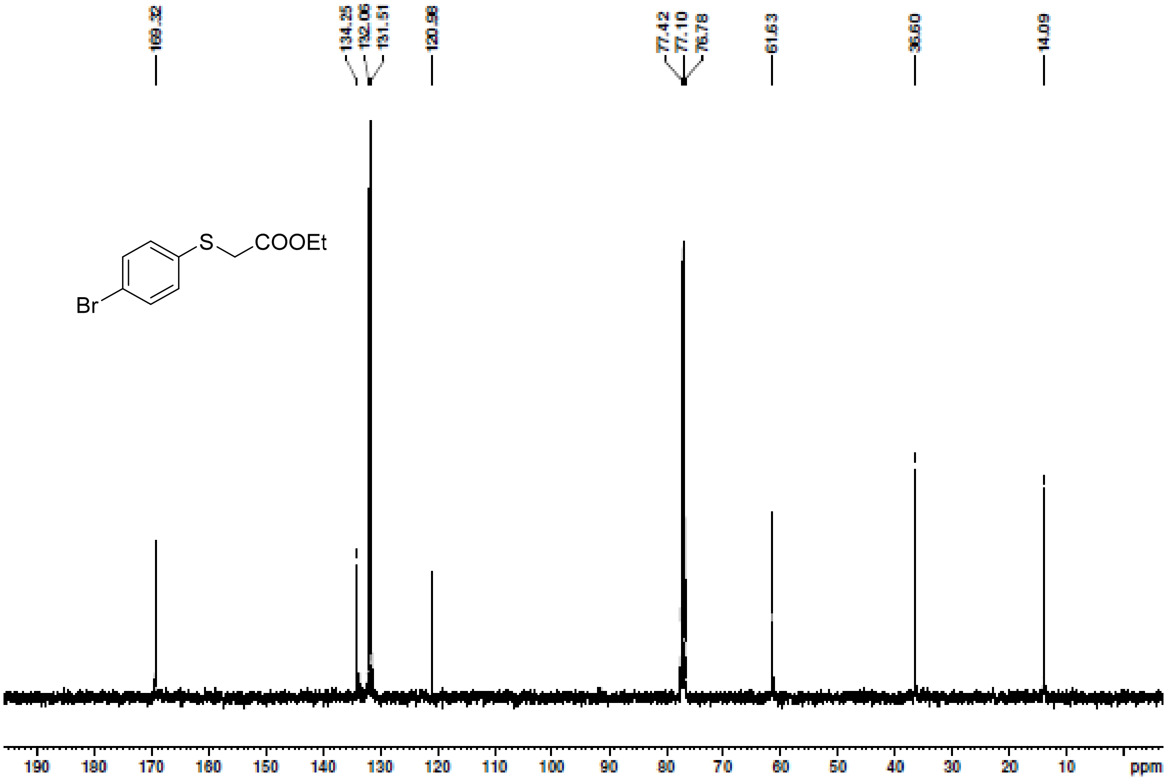
169.32
134.25
132.05
131.51
120.98
77.42
77.10
76.78
61.63
36.60
14.09
S
COOEt
Br
190 180 170 160 150 140 130 120 110 100 90 80 70 60 50 40 30 20 10 ppm

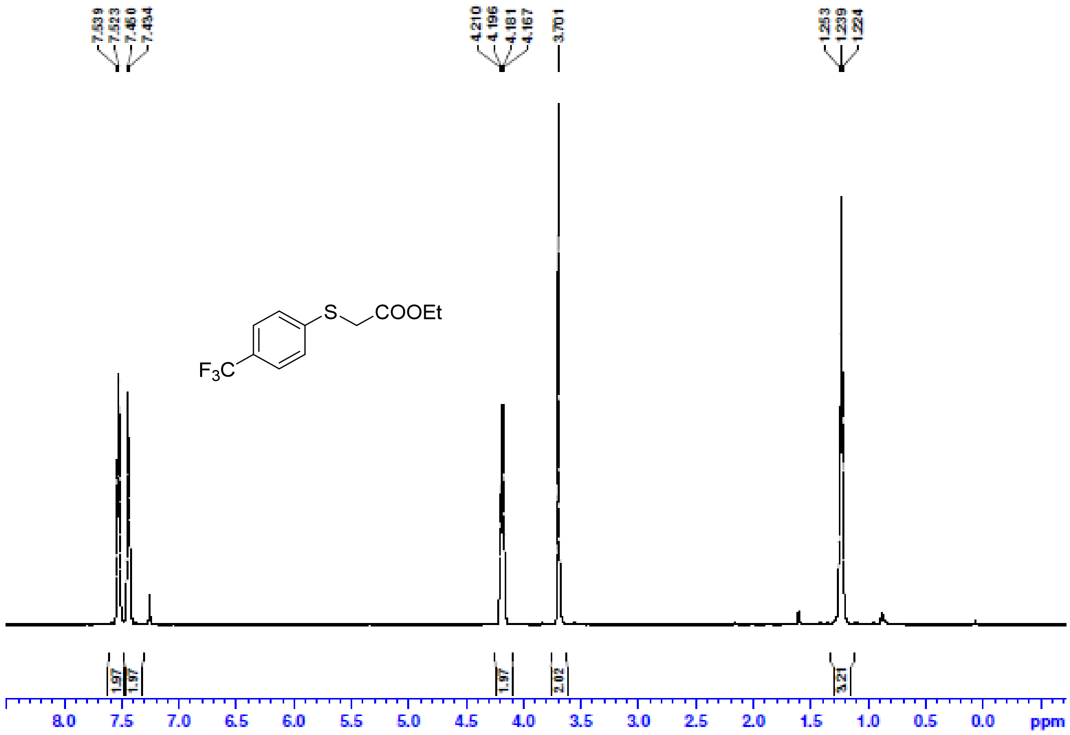
7.539
7.523
7.450
7.434
4.210
4.196
4.181
4.167
3.701
1.253
1.239
1.224
S
COOEt
F_3C
1.97
1.97
1.97
2.02
3.21
8.0 7.5 7.0 6.5 6.0 5.5 5.0 4.5 4.0 3.5 3.0 2.5 2.0 1.5 1.0 0.5 0.0 ppm

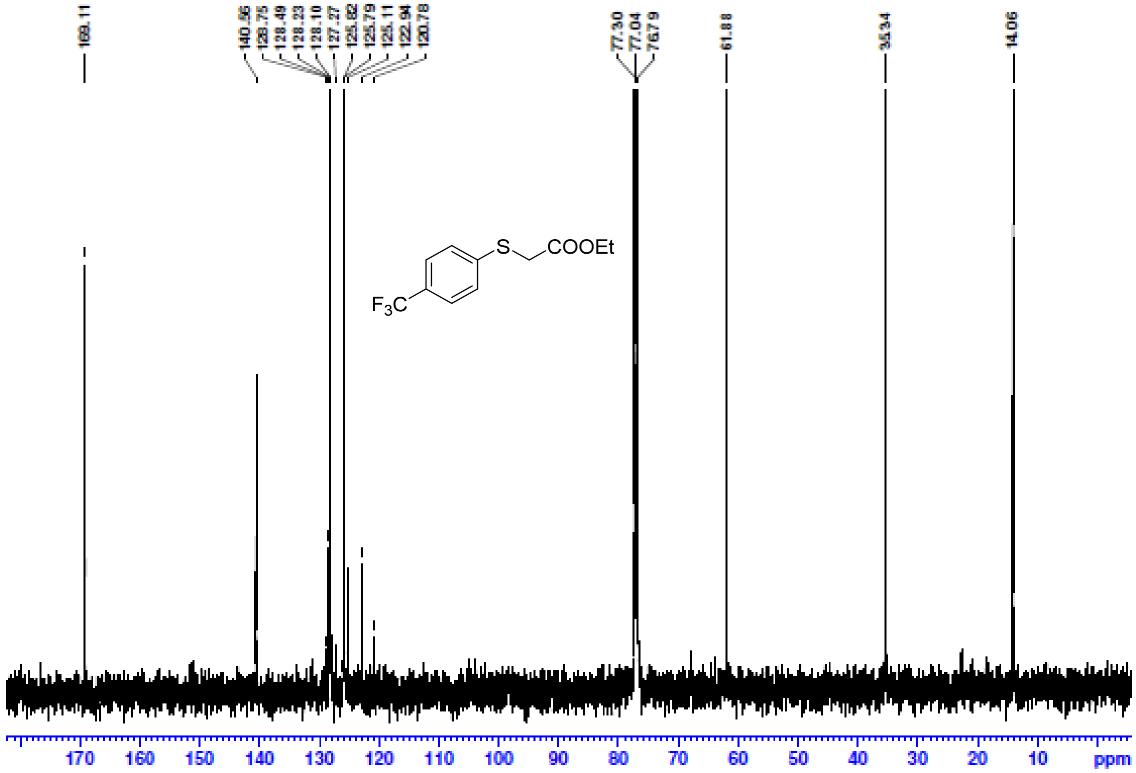
169.11
140.56
128.75
128.49
128.23
128.10
127.27
125.82
125.79
125.11
122.94
120.78
77.30
77.04
76.79
61.88
35.34
14.06
S
COOEt
F_3C
170 160 150 140 130 120 110 100 90 80 70 60 50 40 30 20 10 ppm

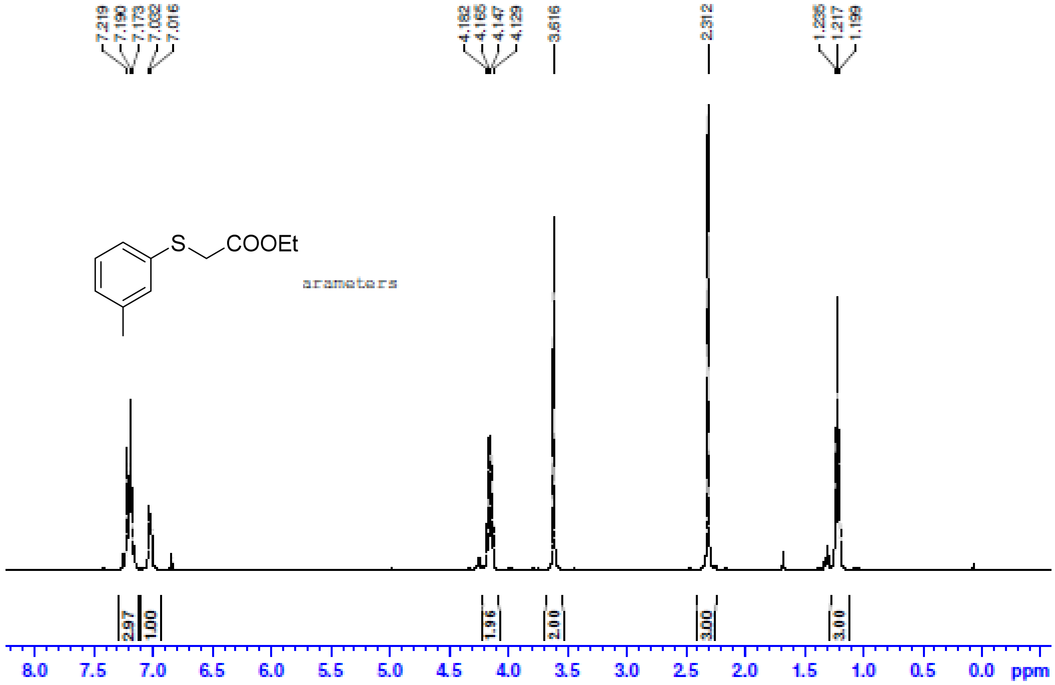
7.219
7.190
7.173
7.032
7.016
4.182
4.165
4.147
4.129
3.616
2.312
1.235
1.217
1.199
S
COOEt
arameters
2.97
1.00
1.96
2.00
3.00
3.00
8.0 7.5 7.0 6.5 6.0 5.5 5.0 4.5 4.0 3.5 3.0 2.5 2.0 1.5 1.0 0.5 0.0 ppm

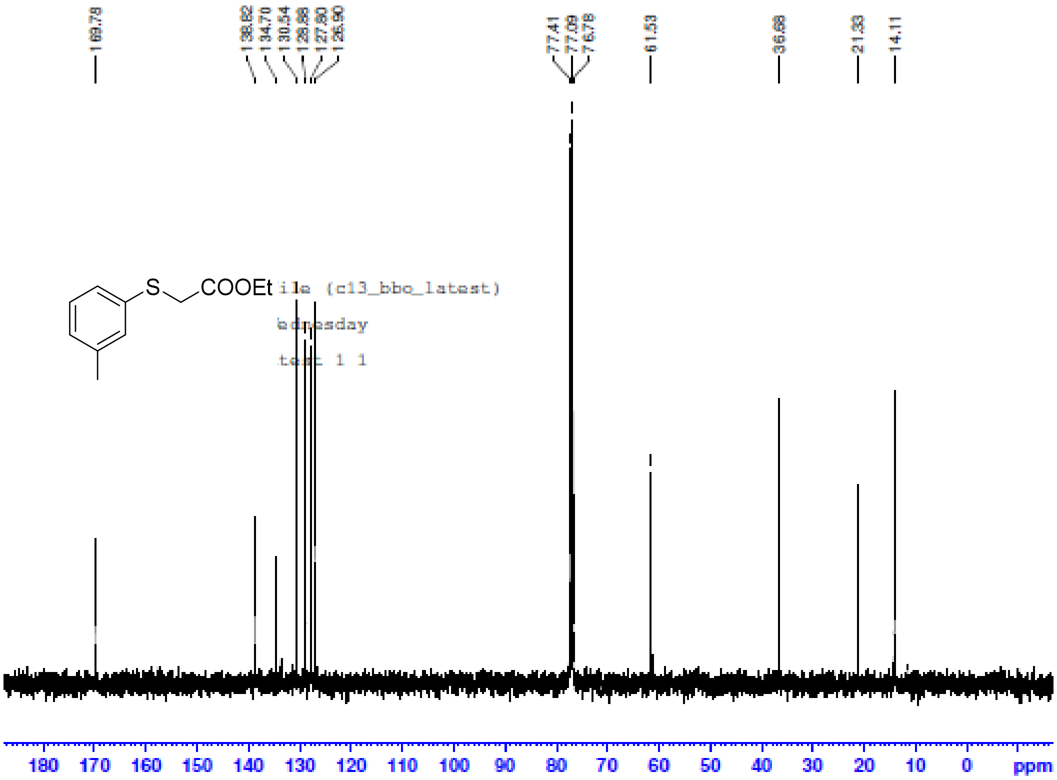
169.78
138.82
134.70
130.54
128.88
127.80
126.90
77.41
77.09
76.78
61.53
36.68
21.33
14.11
S
COOEt
180 170 160 150 140 130 120 110 100 90 80 70 60 50 40 30 20 10 0 ppm

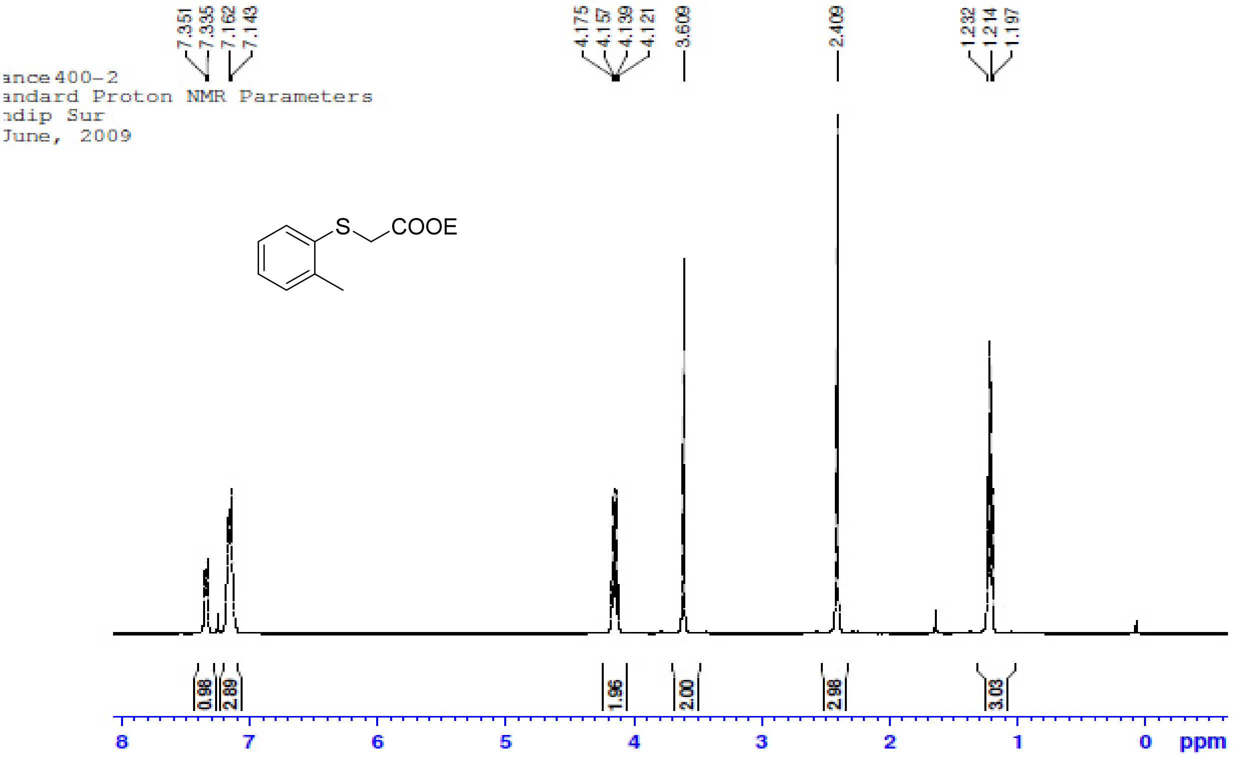
7.351
7.335
7.162
7.143
4.175
4.157
4.139
4.121
3.609
2.409
1.232
1.214
1.197
ance 400-2
andard Proton NMR Parameters
ndip Sur
June, 2009
S
COOE
0.98
2.89
1.96
2.00
2.98
3.03
8
7
6
5
4
3
2
1
0
ppm

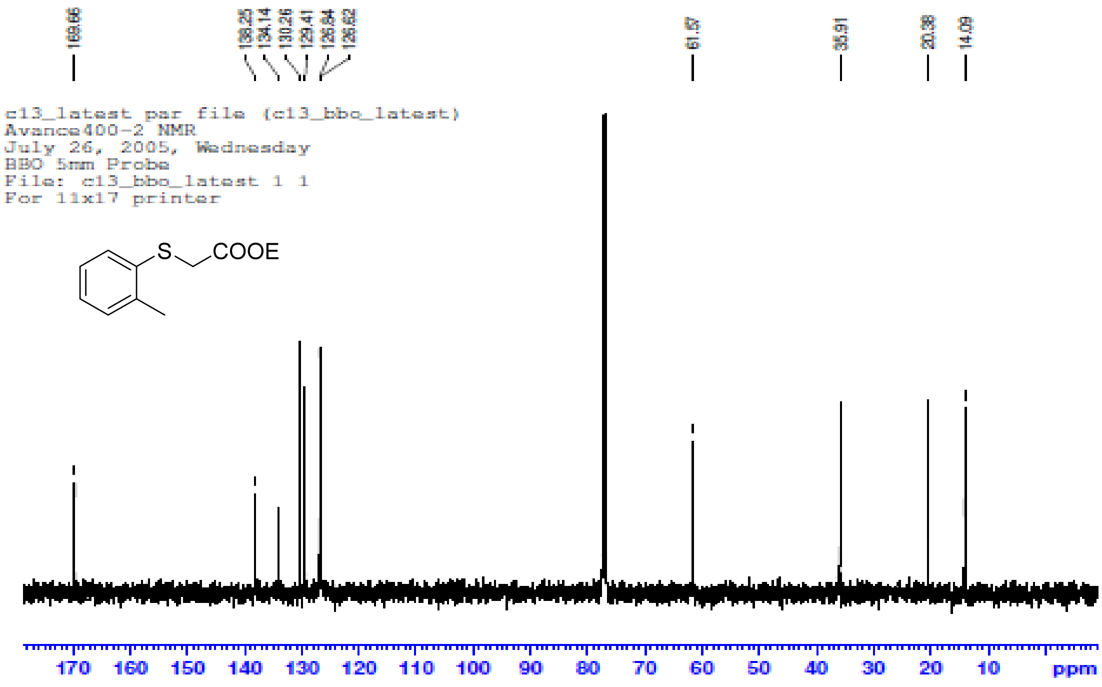
169.66
138.25
134.14
130.26
129.41
126.84
126.62
61.57
35.91
20.38
14.09
c13_latest par file (c13_bbo_latest)
Avance400-2 NMR
July 26, 2005, Wednesday
BBO 5mm Probe
File: c13_bbo_latest 1 1
For 11x17 printer
S
COOE
170
160
150
140
130
120
110
100
90
80
70
60
50
40
30
20
10
ppm

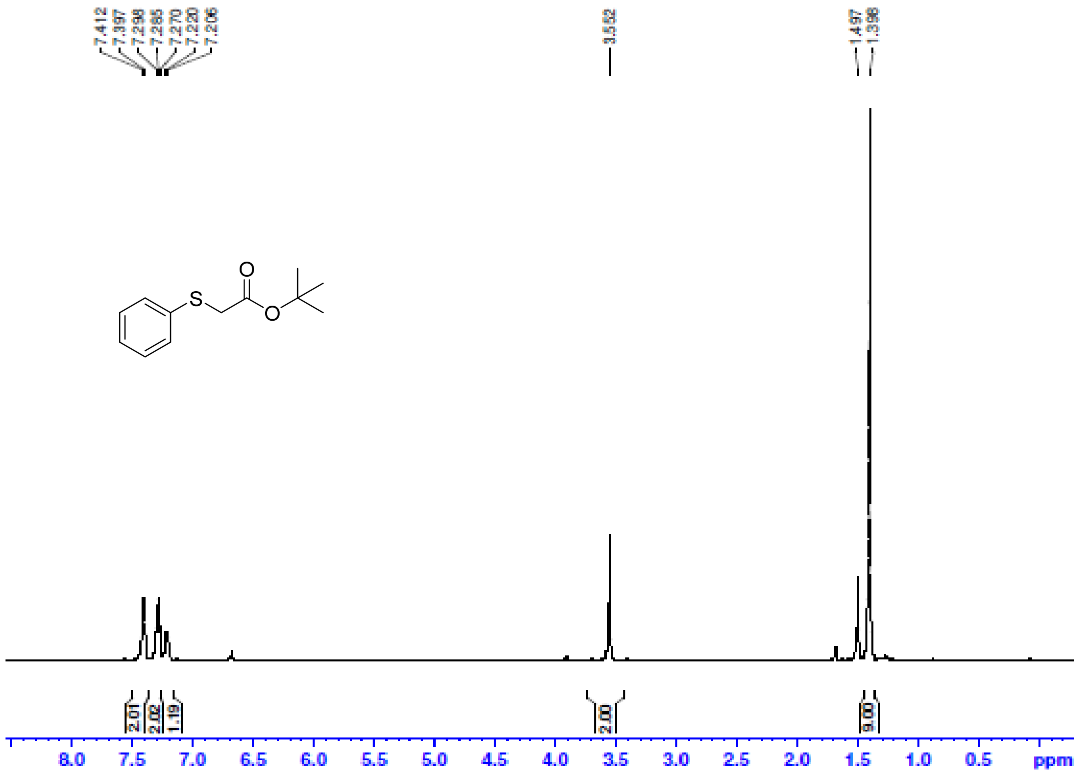
7.412
7.397
7.298
7.285
7.270
7.220
7.206
3.552
1.497
1.398
S
O
O
2.01
2.02
1.19
2.00
9.00
8.0
7.5
7.0
6.5
6.0
5.5
5.0
4.5
4.0
3.5
3.0
2.5
2.0
1.5
1.0
0.5
ppm

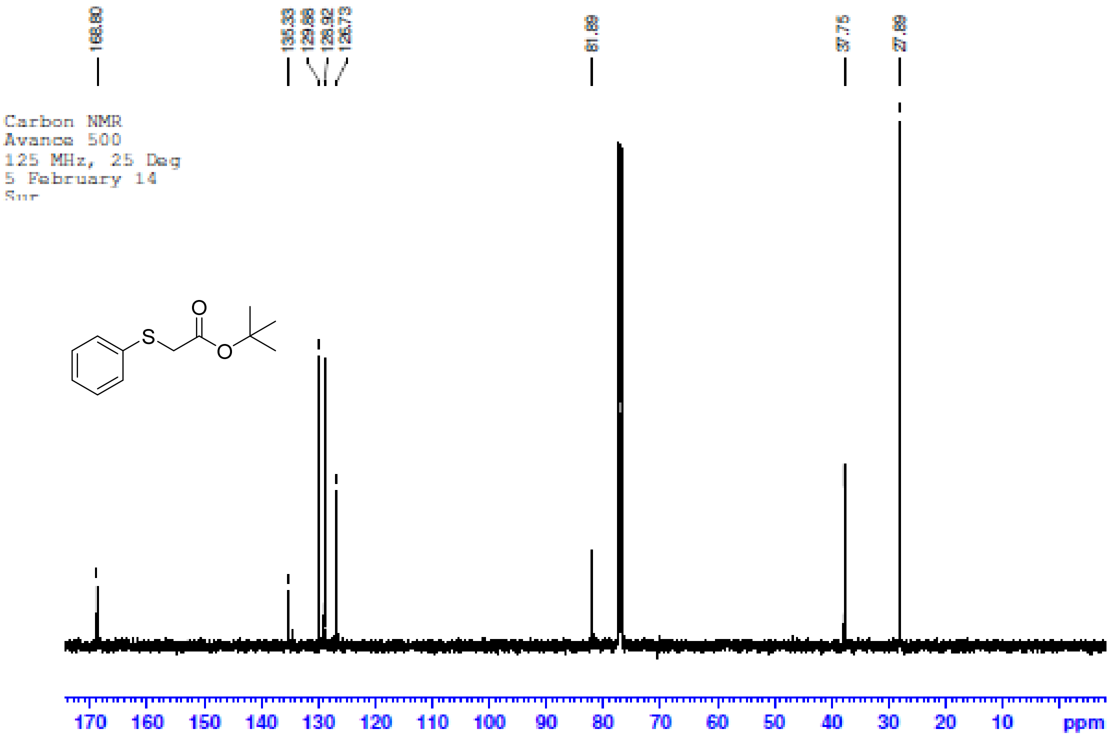
168.80
135.33
129.86
128.92
126.73
81.89
37.75
27.89
Carbon NMR
Avance 500
125 MHz, 25 Deg
5 February 14
S
O
O
170
160
150
140
130
120
110
100
90
80
70
60
50
40
30
20
10
ppm

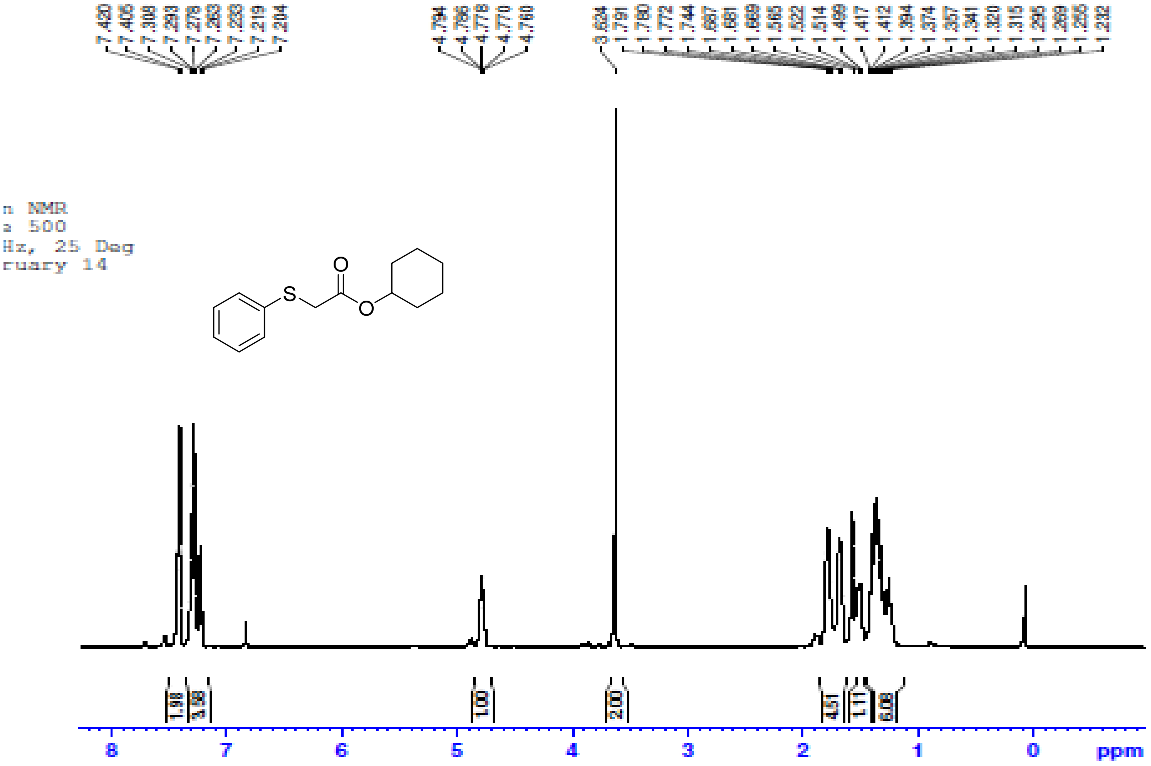
7.420
7.405
7.308
7.293
7.278
7.263
7.233
7.219
7.204
4.794
4.786
4.778
4.770
4.760
3.624
1.791
1.780
1.772
1.744
1.687
1.681
1.669
1.565
1.522
1.514
1.499
1.417
1.412
1.394
1.374
1.357
1.341
1.320
1.315
1.295
1.269
1.255
1.232
n NMR
z 500
Hz, 25 Deg
ruary 14
S
O
O
1.98
3.38
1.00
2.00
4.51
1.11
6.08
8 7 6 5 4 3 2 1 0 ppm

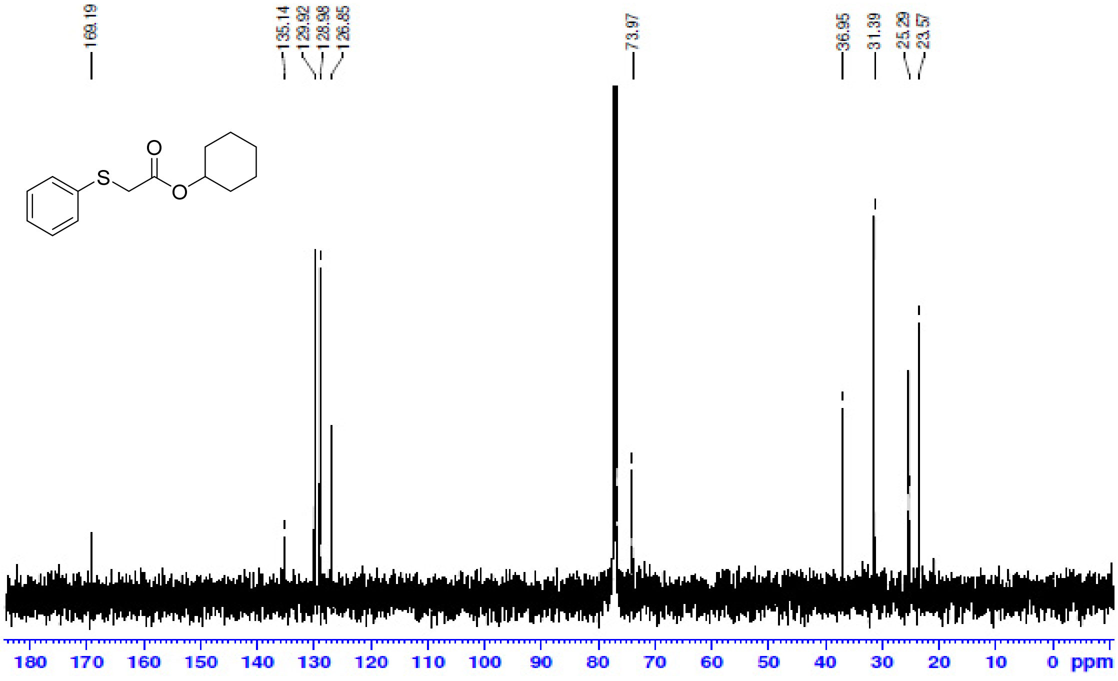
169.19
135.14
129.92
128.98
126.85
73.97
36.95
31.39
25.29
23.57
S
O
O
180 170 160 150 140 130 120 110 100 90 80 70 60 50 40 30 20 10 0 ppm

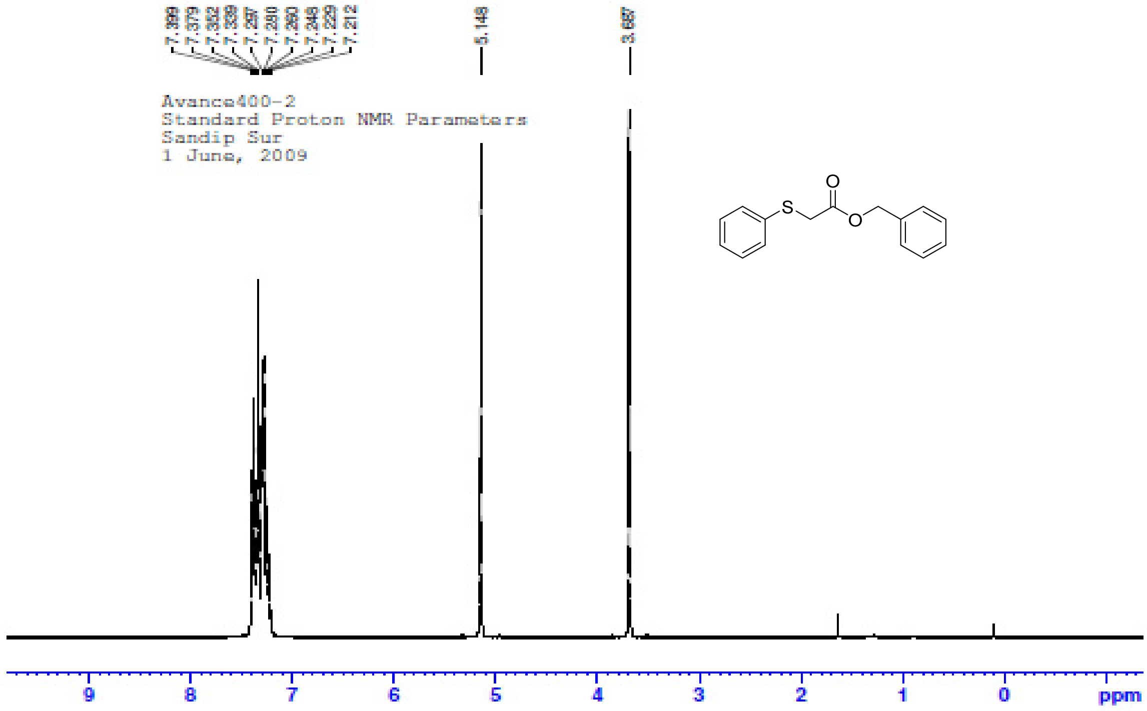

7.399
7.379
7.352
7.339
7.297
7.280
7.260
7.248
7.229
7.212
5.148
3.687
Avance400-2
Standard Proton NMR Parameters
Sandip Sur
1 June, 2009
S
O
O
9
8
7
6
5
4
3
2
1
0
ppm

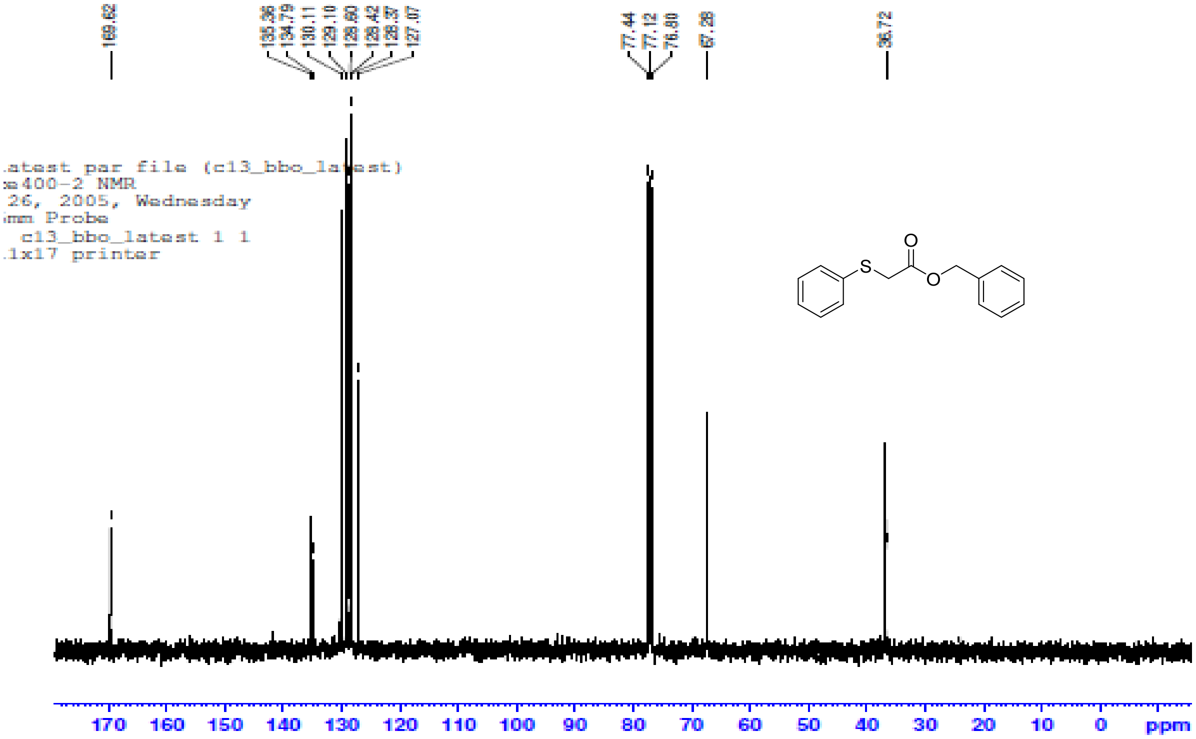

169.62
135.36
134.79
130.11
129.10
128.60
128.42
128.37
127.07
77.44
77.12
76.80
67.28
36.72
S
O
O
170
160
150
140
130
120
110
100
90
80
70
60
50
40
30
20
10
0
ppm

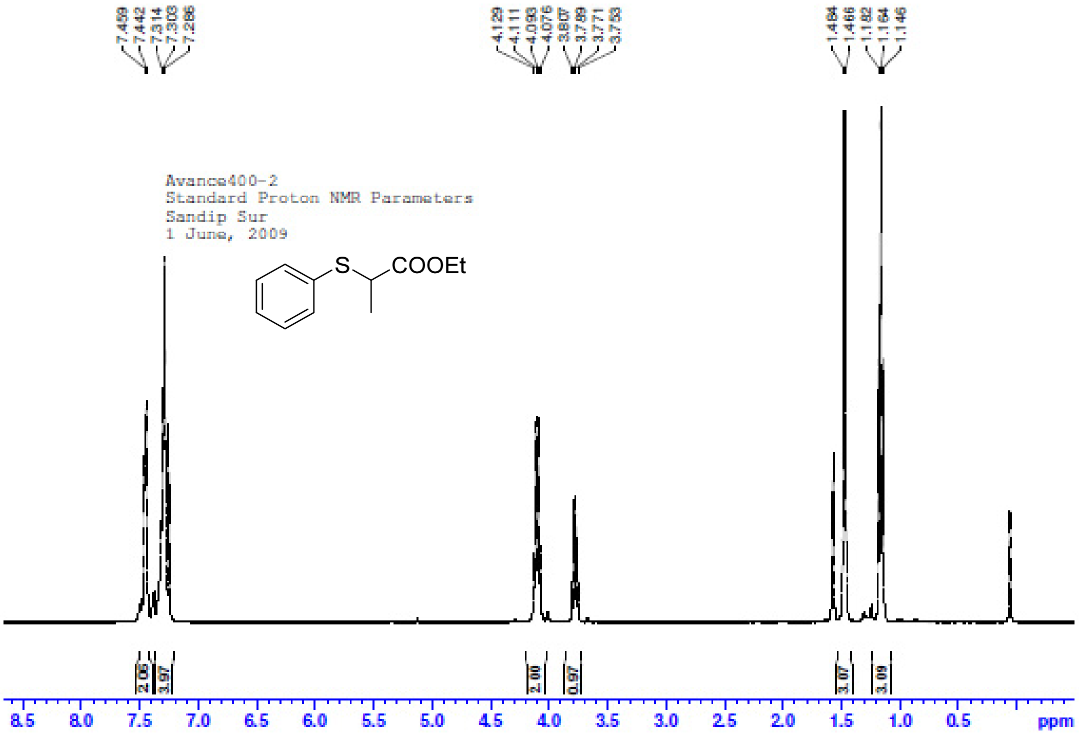
7.459
7.442
7.314
7.303
7.286
4.129
4.111
4.093
4.076
3.807
3.789
3.771
3.753
1.484
1.466
1.182
1.164
1.146
Avance400-2
Standard Proton NMR Parameters
Sandip Sur
1 June, 2009
S
COOEt
2.06
3.97
2.00
0.97
3.07
3.09
8.5 8.0 7.5 7.0 6.5 6.0 5.5 5.0 4.5 4.0 3.5 3.0 2.5 2.0 1.5 1.0 0.5 ppm

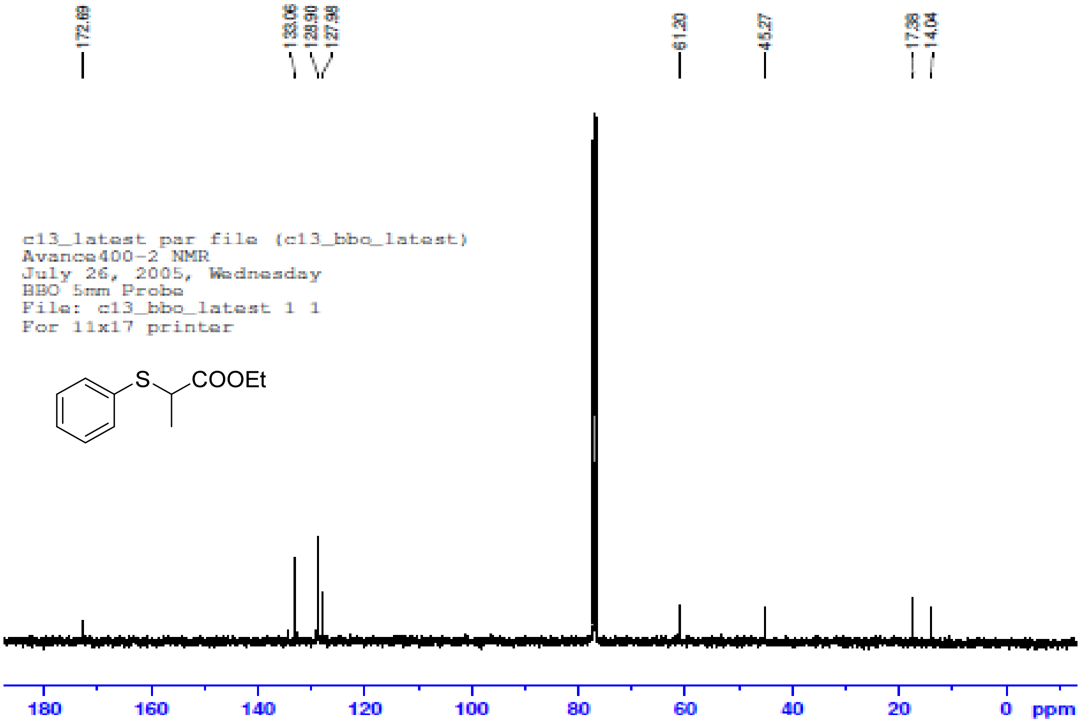
172.69
133.06
128.90
127.98
61.20
45.27
17.38
14.04
c13_latest par file (c13_bbo_latest)
Avance400-2 NMR
July 26, 2005, Wednesday
BBO 5mm Probe
File: c13_bbo_latest 1 1
For 11x17 printer
S
COOEt
180 160 140 120 100 80 60 40 20 0 ppm

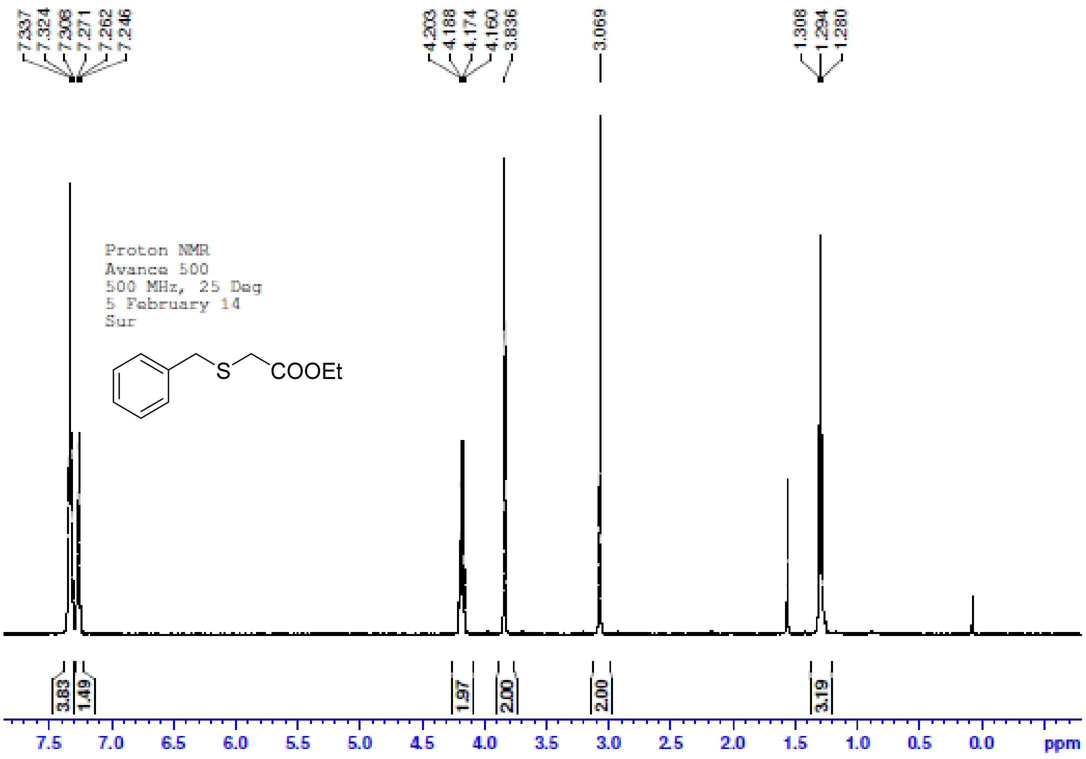
7.337
7.324
7.306
7.271
7.262
7.246
4.203
4.188
4.174
4.160
3.836
3.069
1.308
1.294
1.280
Proton NMR
Avance 500
500 MHz, 25 Deg
5 February 14
Sur
S
COOEt
3.83
1.49
1.97
2.00
2.00
3.19
7.5 7.0 6.5 6.0 5.5 5.0 4.5 4.0 3.5 3.0 2.5 2.0 1.5 1.0 0.5 0.0 ppm

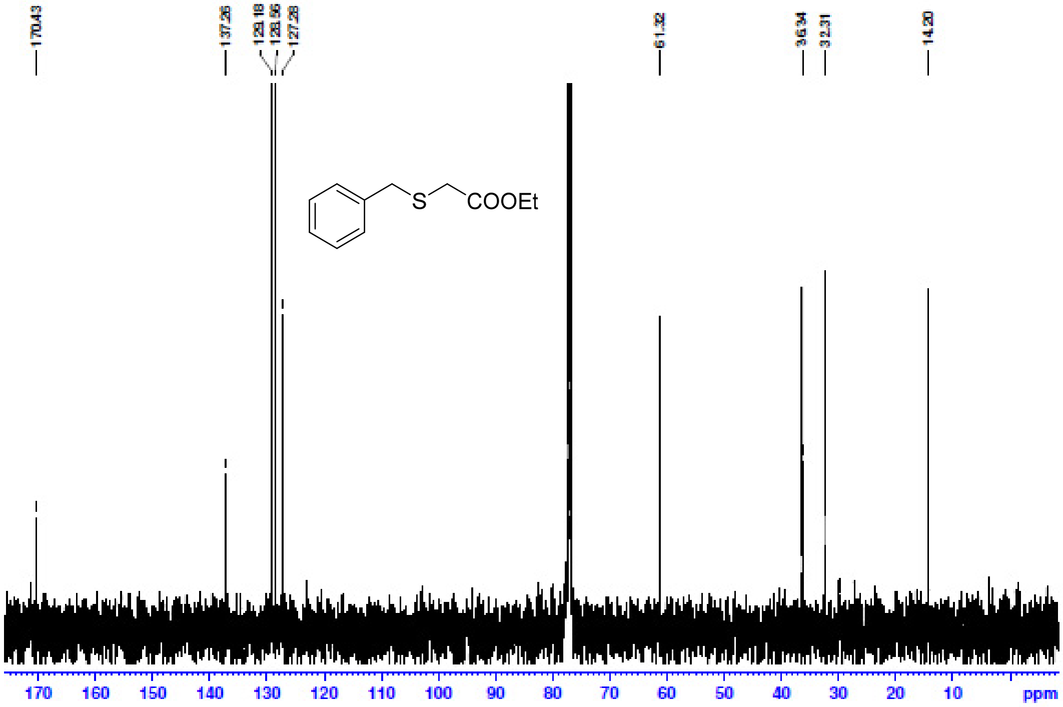
170.43
137.26
129.18
128.56
127.28
61.32
36.34
32.31
14.20
S
COOEt
170 160 150 140 130 120 110 100 90 80 70 60 50 40 30 20 10 ppm

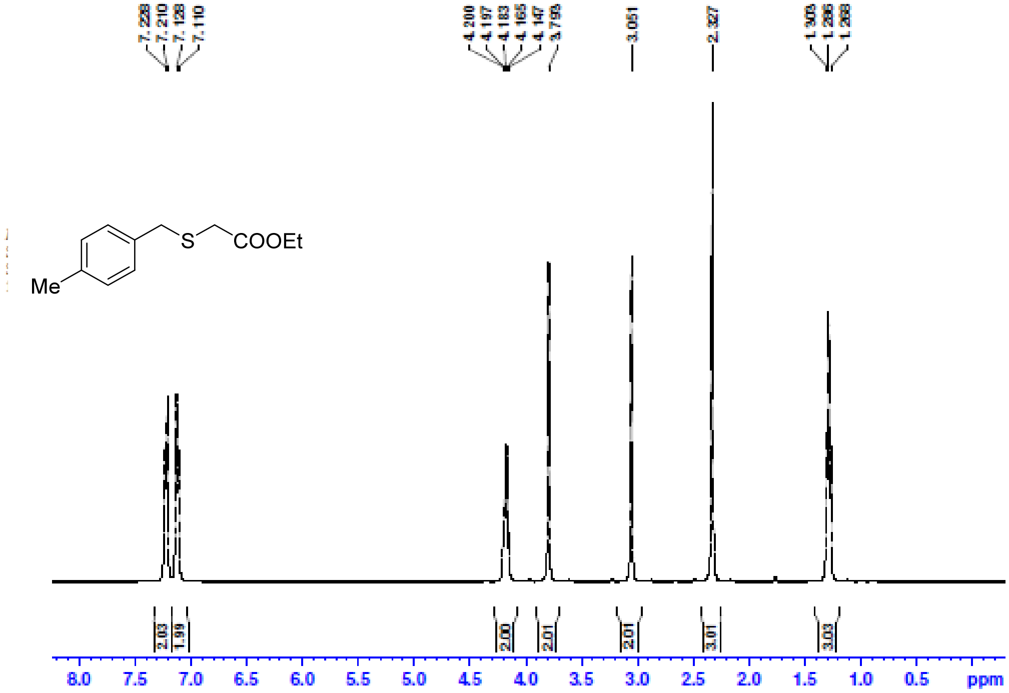
7.228
7.210
7.128
7.110
4.200
4.197
4.183
4.165
4.147
3.793
3.051
2.327
1.303
1.286
1.268
S
COOEt
Me
2.03
1.99
2.00
2.01
2.01
3.01
3.03
ppm

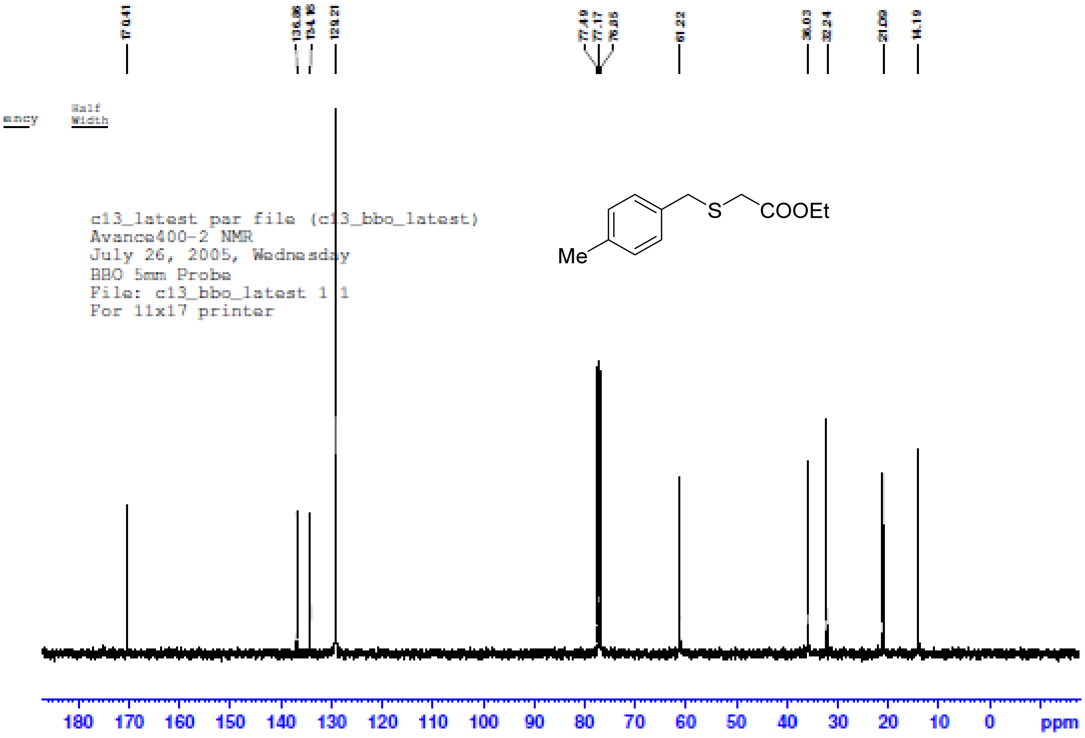
c13_latest par file (c13_bbo_latest)
Avance400-2 NMR
July 26, 2005, Wednesday
BBO 5mm Probe
File: c13_bbo_latest 1 1
For 11x17 printer
S
COOEt
Me
ppm

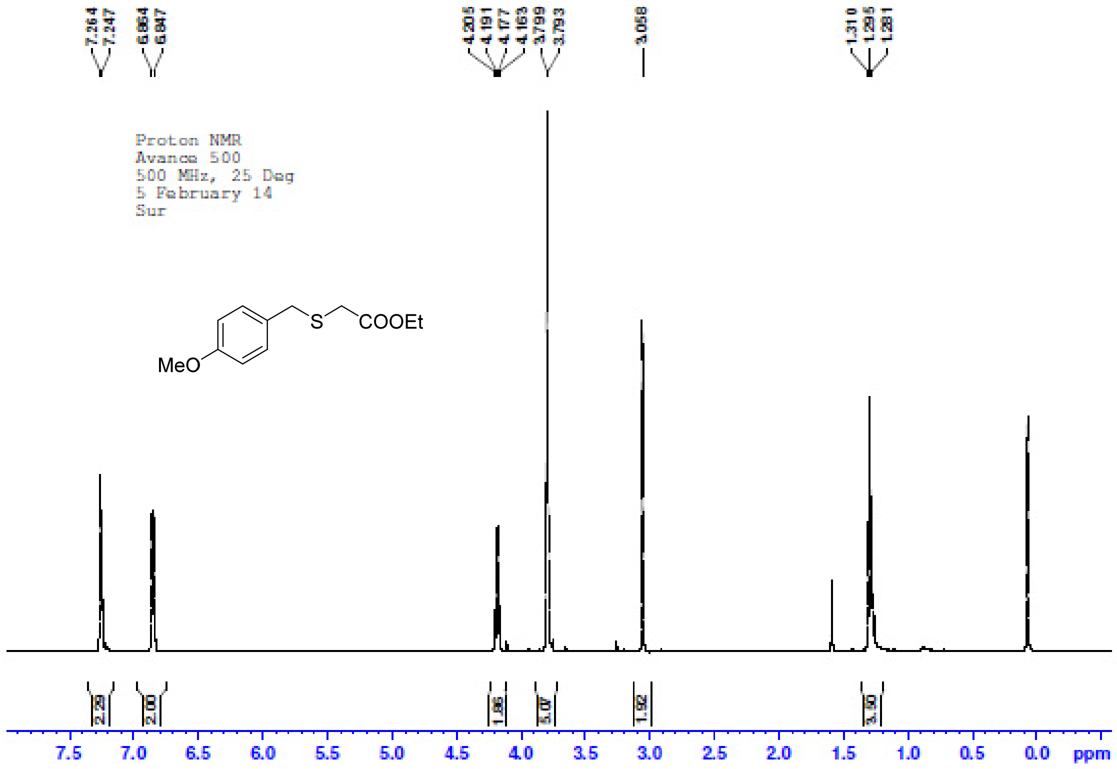
7.264
7.247
6.864
6.847
4.205
4.191
4.177
4.163
3.799
3.793
3.058
1.310
1.295
1.281
Proton NMR
Avance 500
500 MHz, 25 Deg
5 February 14
Sur
MeO
S
COOEt
2.29
2.00
1.86
5.07
1.92
3.50
ppm

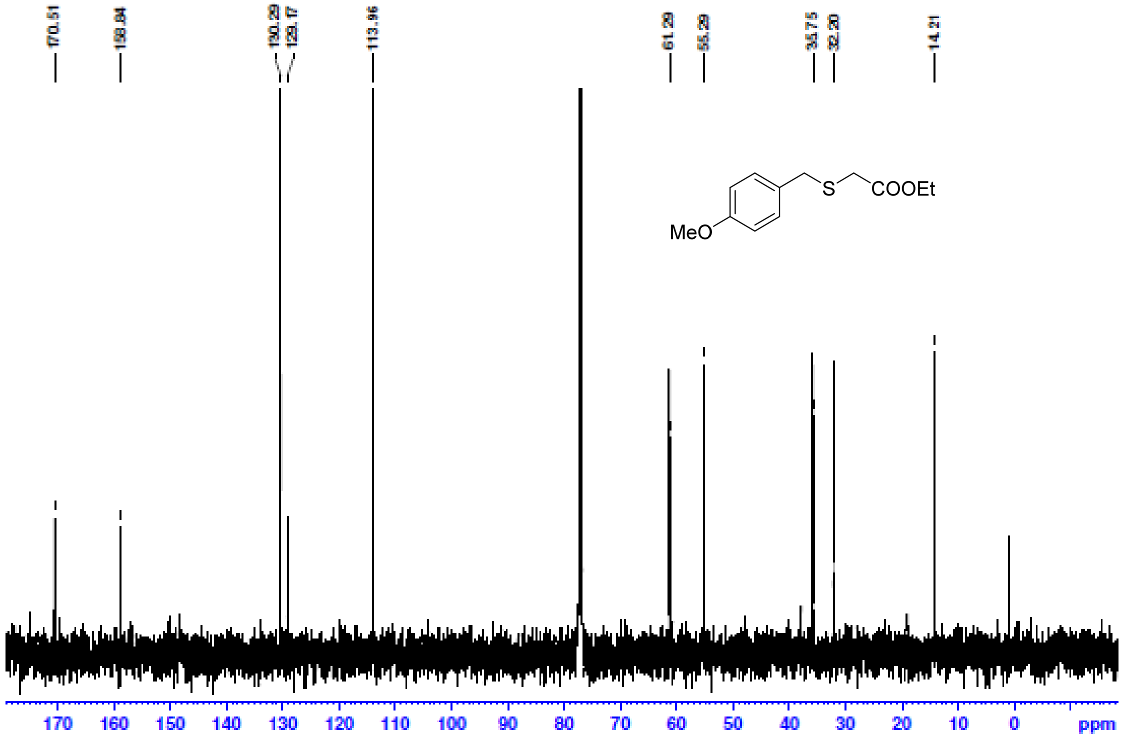
170.51
158.84
130.29
129.17
113.96
61.29
55.29
35.75
32.20
14.21
MeO
S
COOEt
ppm

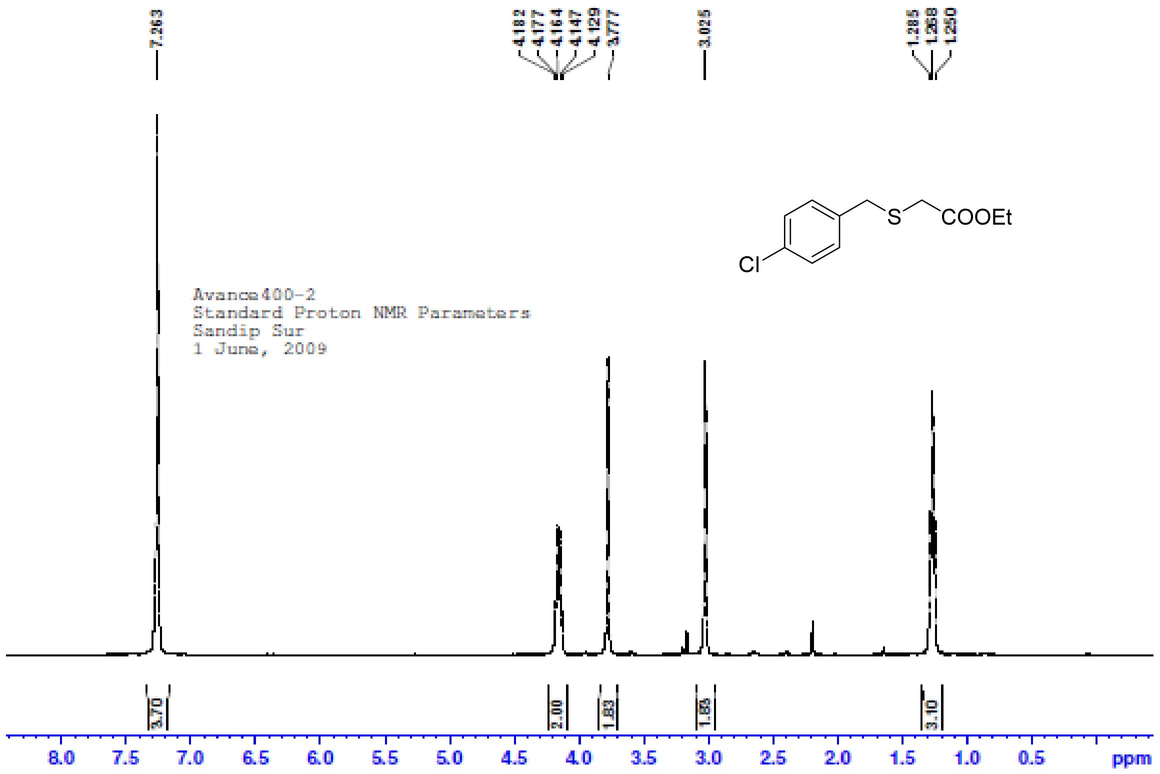
7.263
4.182
4.177
4.164
4.147
4.129
3.777
3.025
1.285
1.268
1.250
COOEt
S
Cl
Avance400-2
Standard Proton NMR Parameters
Sandip Sur
1 June, 2009
3.70
2.00
1.83
1.85
3.10
8.0 7.5 7.0 6.5 6.0 5.5 5.0 4.5 4.0 3.5 3.0 2.5 2.0 1.5 1.0 0.5 ppm

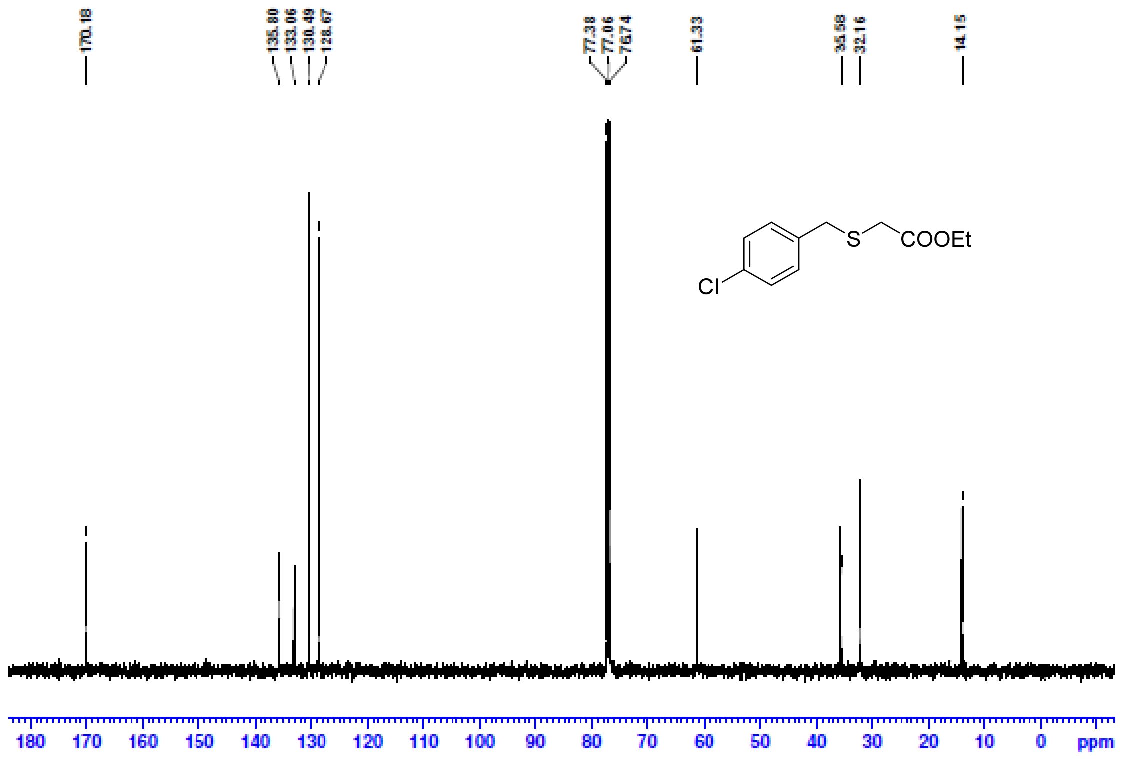
170.18
135.80
133.06
130.49
128.67
77.38
77.06
76.74
61.33
35.58
32.16
14.15
COOEt
S
Cl
180 170 160 150 140 130 120 110 100 90 80 70 60 50 40 30 20 10 0 ppm

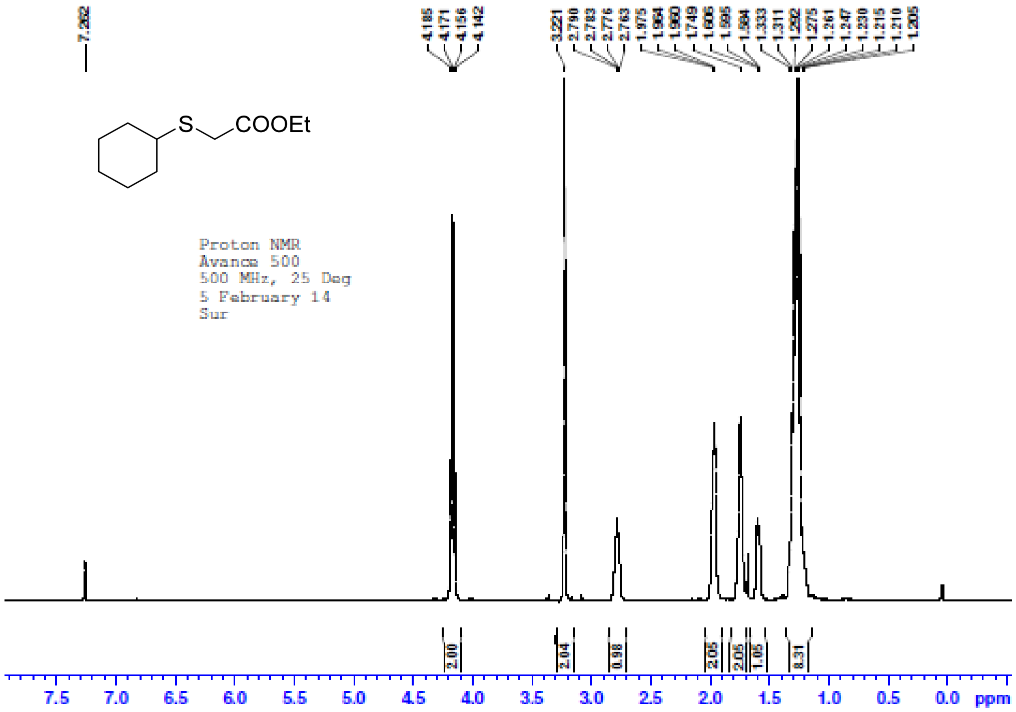
S
COOEt
Proton NMR
Avance 500
500 MHz, 25 Deg
5 February 14
Sur
ppm

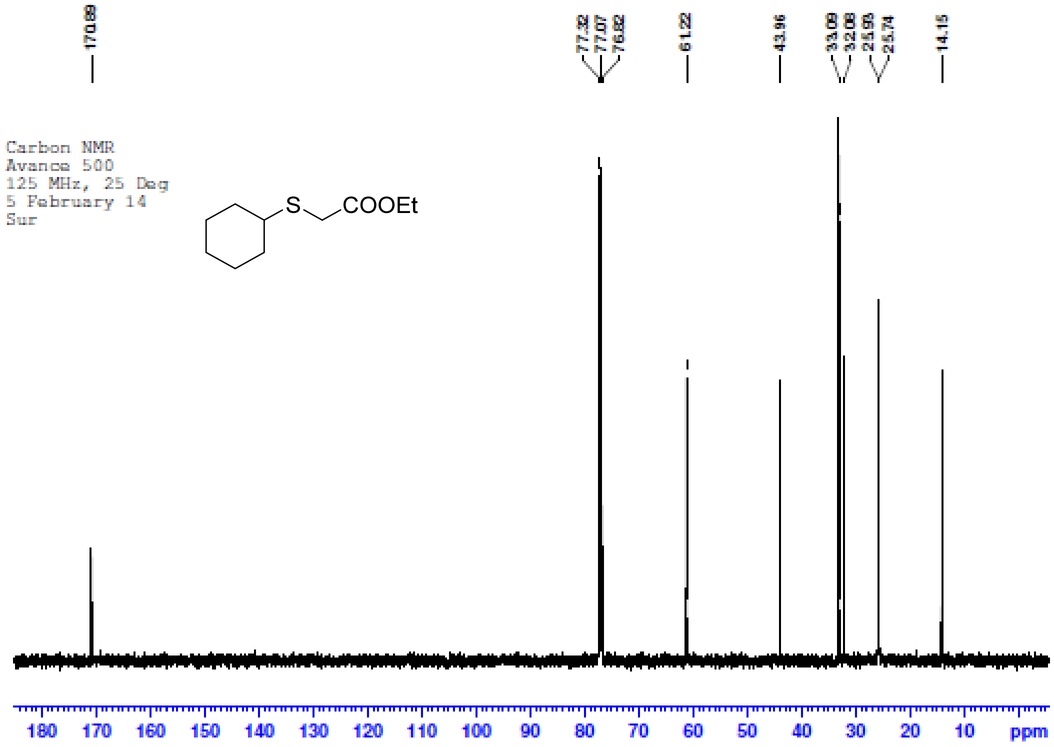
Carbon NMR
Avance 500
125 MHz, 25 Deg
5 February 14
Sur
S
COOEt
170.89
77.32
77.07
76.82
61.22
43.96
33.09
32.08
25.93
25.74
14.15
ppm

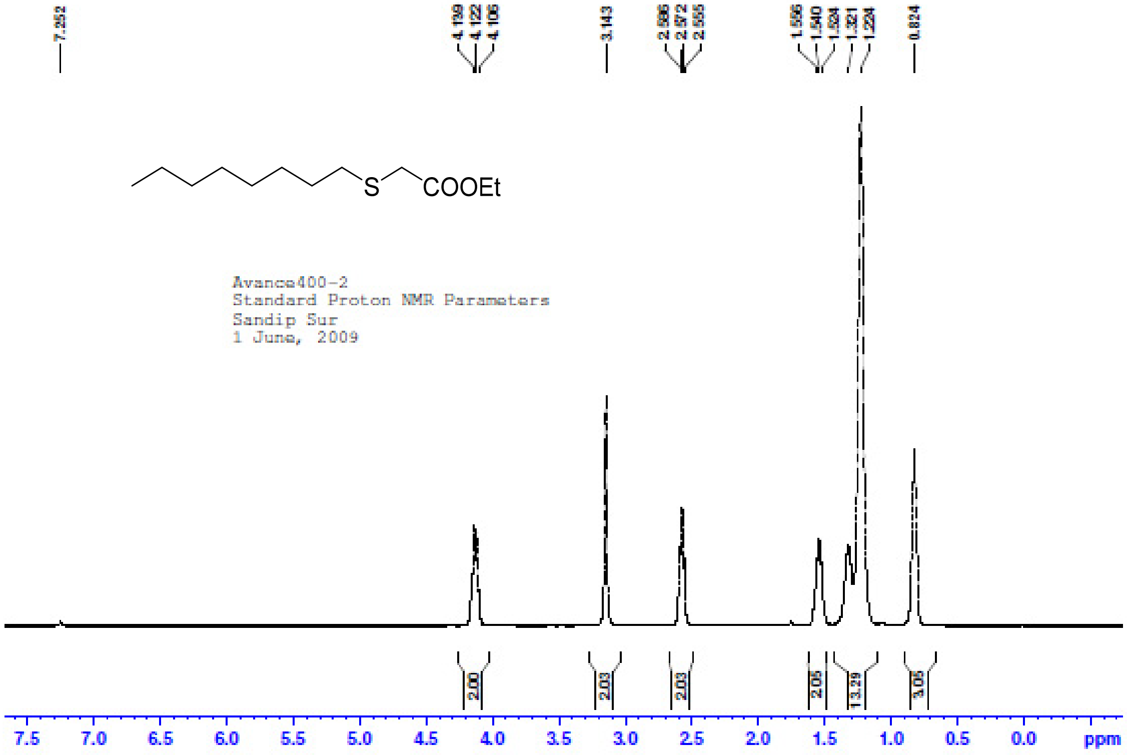
7.252
4.139
4.122
4.106
3.143
2.586
2.572
2.555
1.556
1.540
1.524
1.321
1.224
0.824
S
COOEt
Avance400-2
Standard Proton NMR Parameters
Sandip Sur
1 June, 2009
2.00
2.03
2.03
2.05
13.29
3.05
7.5
7.0
6.5
6.0
5.5
5.0
4.5
4.0
3.5
3.0
2.5
2.0
1.5
1.0
0.5
0.0
ppm

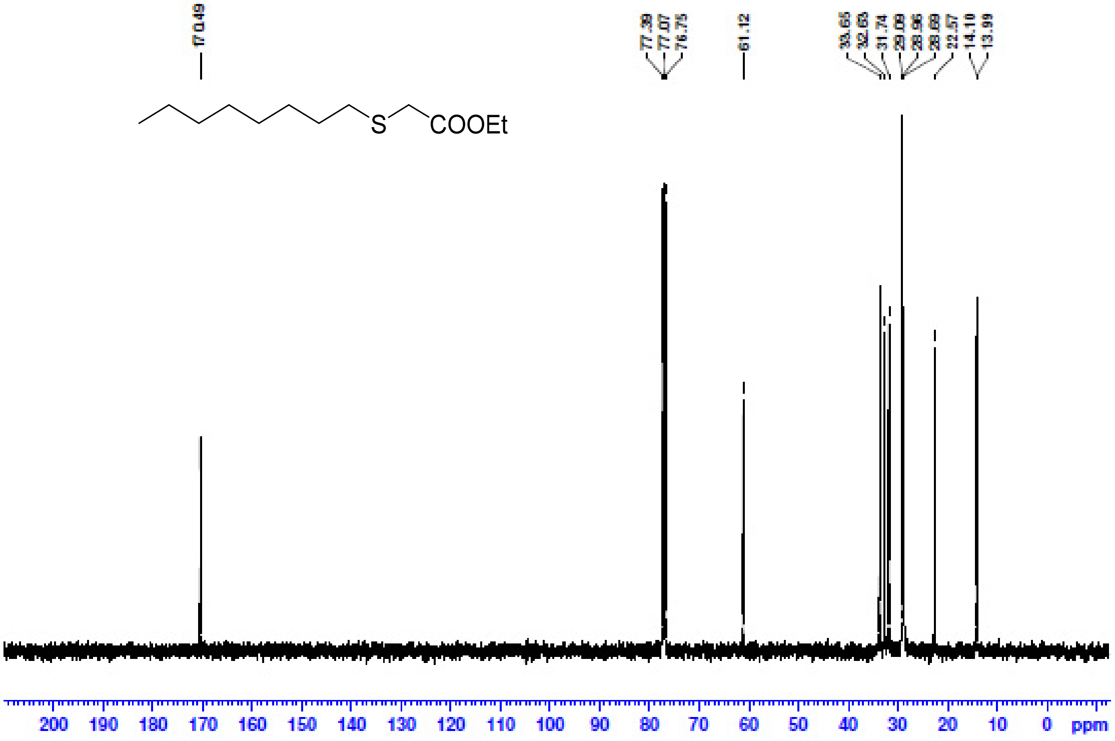
170.49
77.39
77.07
76.75
61.12
33.65
32.63
31.74
29.09
28.96
28.69
22.57
14.10
13.99
S
COOEt
200
190
180
170
160
150
140
130
120
110
100
90
80
70
60
50
40
30
20
10
0
ppm

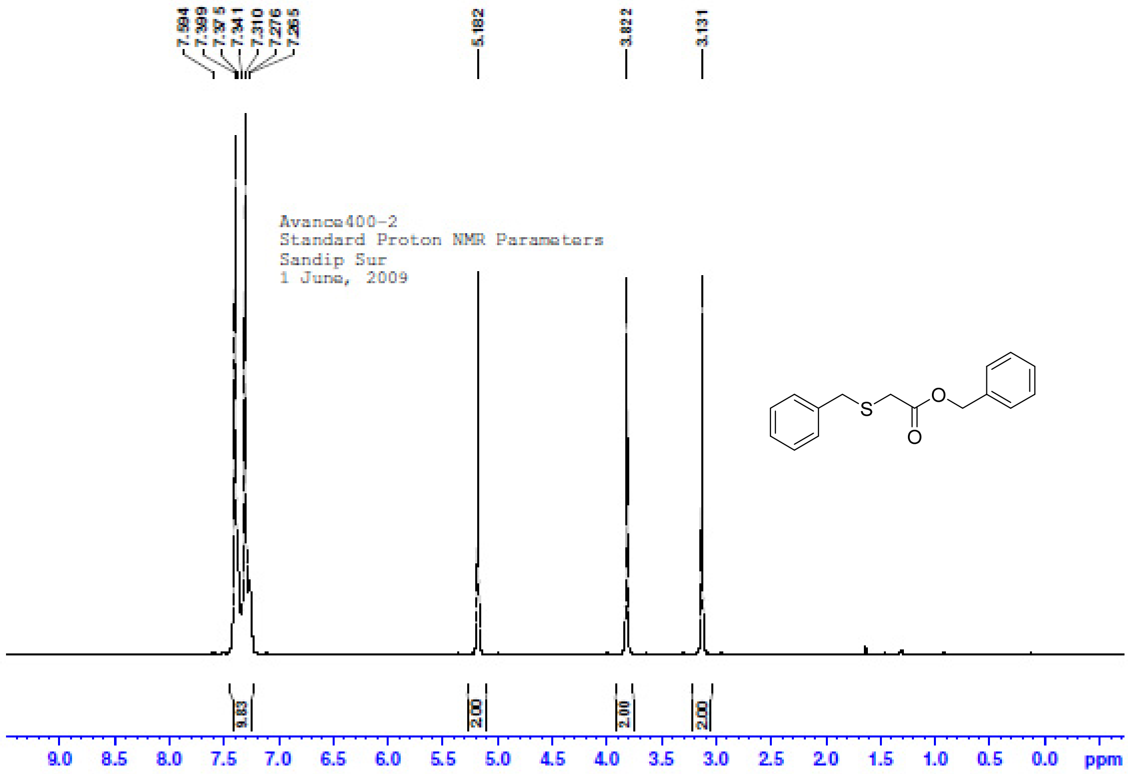
7.594
7.399
7.375
7.341
7.310
7.276
7.265
5.182
3.822
3.131
Avance400-2
Standard Proton NMR Parameters
Sandip Sur
1 June, 2009
S
O
O
9.83
2.00
2.00
2.00
9.0 8.5 8.0 7.5 7.0 6.5 6.0 5.5 5.0 4.5 4.0 3.5 3.0 2.5 2.0 1.5 1.0 0.5 0.0 ppm

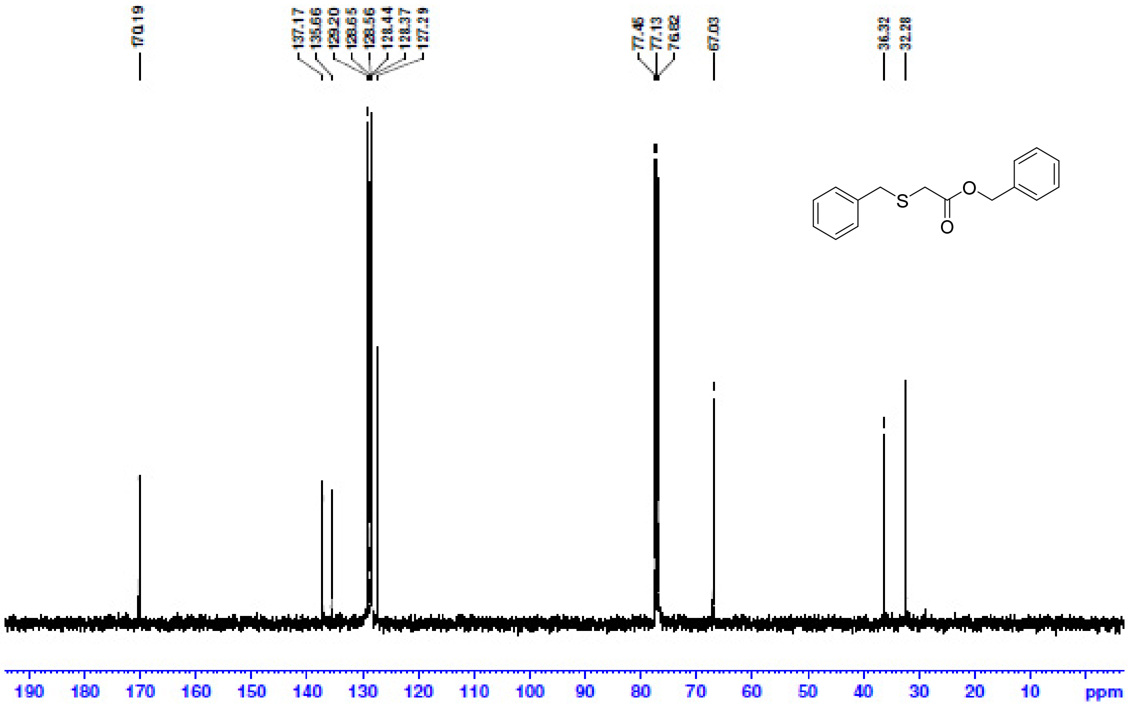
170.19
137.17
135.66
129.20
128.65
128.56
128.44
128.37
127.29
77.45
77.13
76.82
67.03
36.32
32.28
S
O
O
190 180 170 160 150 140 130 120 110 100 90 80 70 60 50 40 30 20 10 ppm

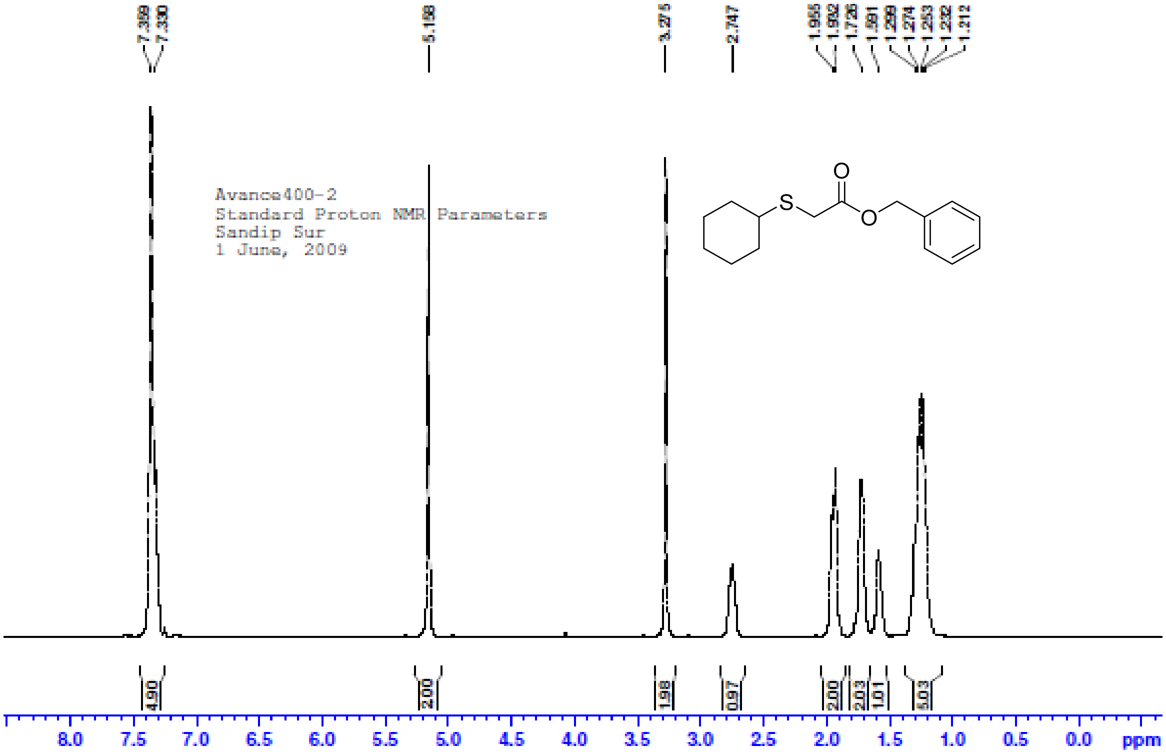
7.359
7.330
5.168
3.275
2.747
1.955
1.932
1.726
1.591
1.299
1.274
1.253
1.232
1.212
Avance400-2
Standard Proton NMR Parameters
Sandip Sur
1 June, 2009
O
S
O
4.90
2.00
1.98
0.97
2.00
2.03
1.01
5.03
8.0 7.5 7.0 6.5 6.0 5.5 5.0 4.5 4.0 3.5 3.0 2.5 2.0 1.5 1.0 0.5 0.0 ppm

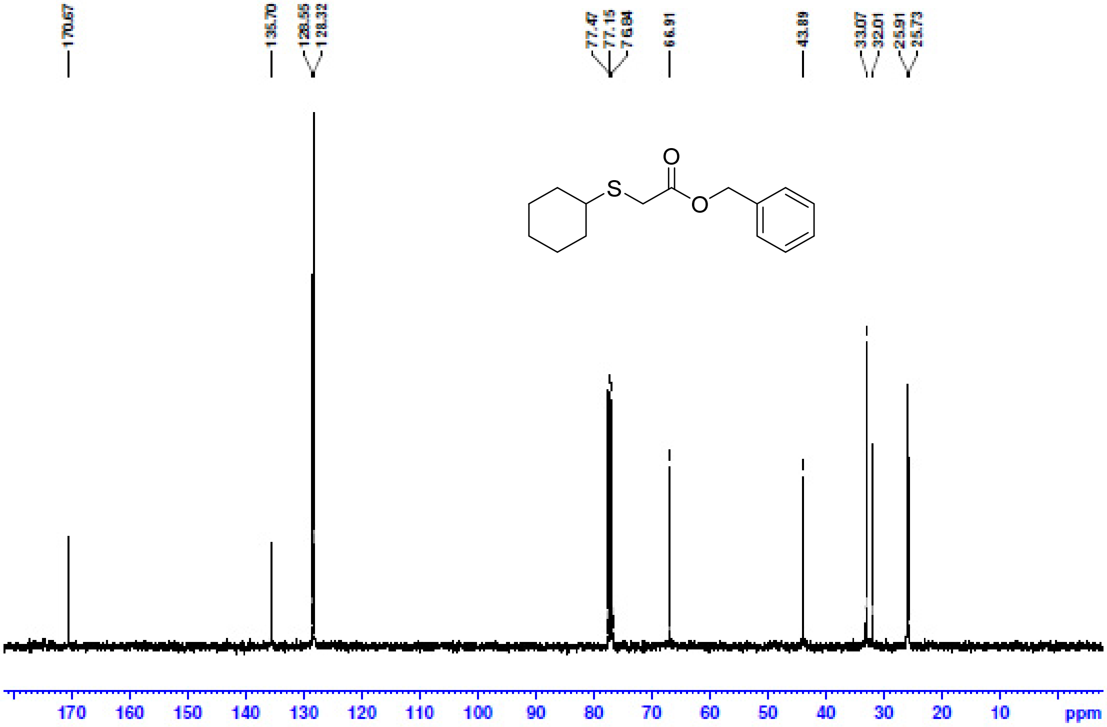
170.67
135.70
128.55
128.32
77.47
77.15
76.84
66.91
43.89
33.07
32.01
25.91
25.73
O
S
O
170 160 150 140 130 120 110 100 90 80 70 60 50 40 30 20 10 ppm

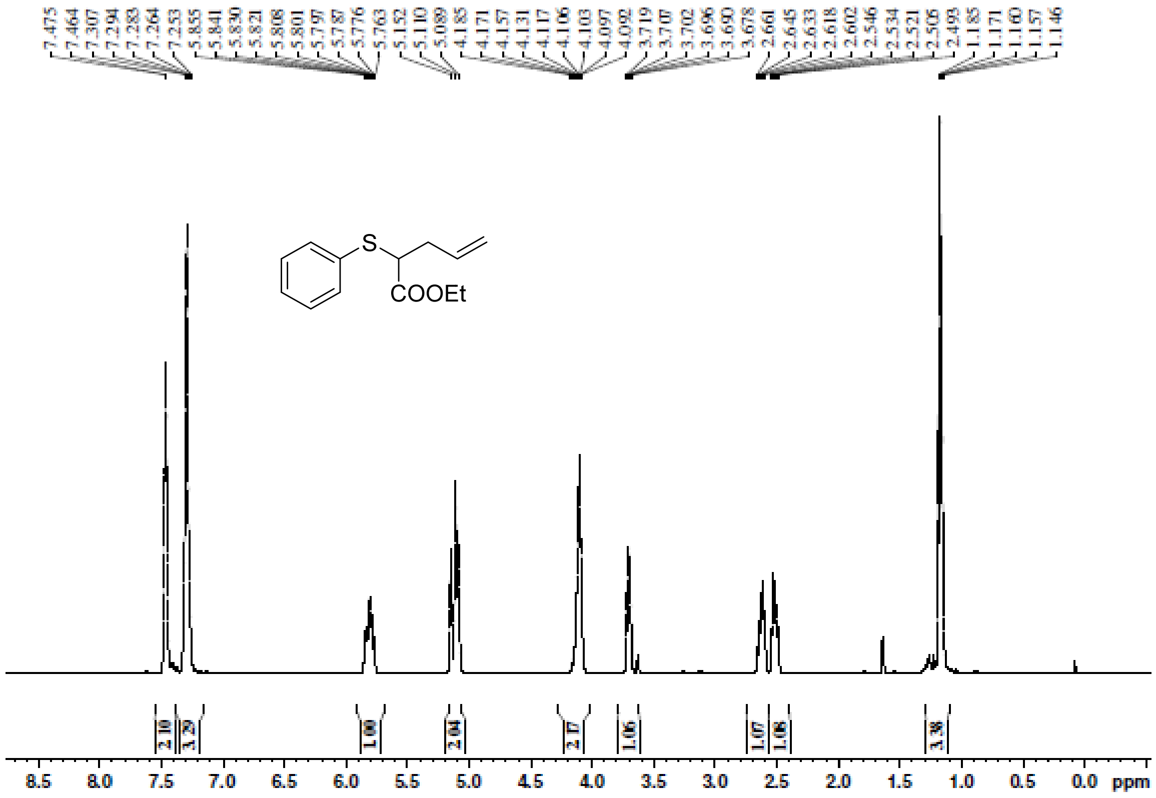
7.475 7.464 7.307 7.294 7.283 7.264 7.253 5.855 5.841 5.830 5.821 5.808 5.801 5.797 5.787 5.776 5.763 5.152 5.110 5.089 4.185 4.171 4.157 4.131 4.117 4.106 4.103 4.097 4.092 3.719 3.707 3.702 3.696 3.690 3.678 2.661 2.645 2.633 2.618 2.602 2.546 2.534 2.521 2.505 2.493 1.183 1.171 1.160 1.157 1.146
S
COOEt
2.10 3.29 1.00 2.04 2.17 1.06 1.07 1.08 3.38
8.5 8.0 7.5 7.0 6.5 6.0 5.5 5.0 4.5 4.0 3.5 3.0 2.5 2.0 1.5 1.0 0.5 0.0 ppm

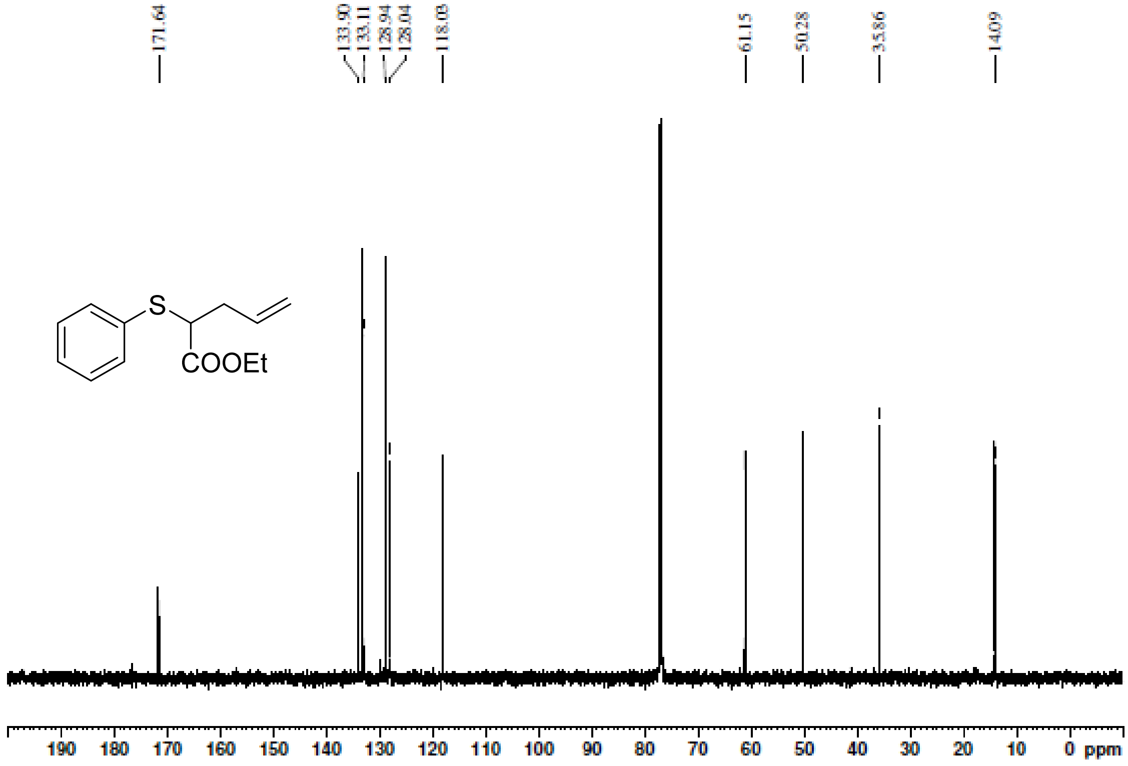
171.64 133.90 133.11 128.94 128.04 118.03 61.15 50.28 35.86 14.09
S
COOEt
190 180 170 160 150 140 130 120 110 100 90 80 70 60 50 40 30 20 10 0 ppm
